# A functional genomics catalogue of activated transcription factors during pathogenesis of pneumococcal disease

**DOI:** 10.1186/1471-2164-15-769

**Published:** 2014-09-08

**Authors:** Layla K Mahdi, Tahereh Deihimi, Fatemeh Zamansani, Mario Fruzangohar, David L Adelson, James C Paton, Abiodun D Ogunniyi, Esmaeil Ebrahimie

**Affiliations:** Research Centre for Infectious Diseases, School of Molecular and Biomedical Science, The University of Adelaide, Adelaide, South Australia Australia; Institute of Biotechnology, Shiraz University, Shiraz, Iran; Australian Centre for Plant Functional Genomics, The University of Adelaide, Waite Campus, Adelaide, South Australia Australia; Centre for Bioinformatics and Computational Genetics, School of Molecular and Biomedical Science, The University of Adelaide, Adelaide, South Australia Australia; School of Molecular and Biomedical Science, The University of Adelaide, Adelaide, South Australia Australia

## Abstract

**Background:**

*Streptococcus pneumoniae* (the pneumococcus) is the world’s foremost microbial pathogen, killing more people each year than HIV, TB or malaria. The capacity to penetrate deeper host tissues contributes substantially to the ability of this organism to cause disease. Here we investigated, for the first time, functional genomics modulation of 3 pneumococcal strains (serotype 2 [D39], serotype 4 [WCH43] and serotype 6A [WCH16]) during transition from the nasopharynx to lungs to blood and to brain of mice at both promoter and domain activation levels.

**Results:**

We found 7 highly activated transcription factors (TFs) [*argR*, *codY*, *hup*, *rpoD*, *rr02*, *scrR* and *smrC*] capable of binding to a large number of up-regulated genes, potentially constituting the regulatory backbone of pneumococcal pathogenesis. Strain D39 showed a distinct profile in employing a large number of TFs during blood infection. Interestingly, the same highly activated TFs used by D39 in blood are also used by WCH16 and WCH43 during brain infection. This indicates that different pneumococcal strains might activate a similar set of TFs and regulatory elements depending on the final site of infection. Hierarchical clustering analysis showed that all the highly activated TFs, except *rpoD*, clustered together with a high level of similarity in all 3 strains, which might suggest redundancy in the regulatory roles of these TFs during infection. Discriminant function analysis of the TFs in various niches highlights differential regulatory backgrounds of the 3 strains, and pathogenesis data confirms *codY* as the most significant predictor discriminating between these strains in various niches, particularly in the blood. Moreover, the predicted TF and domain activation profiles of the 3 strains correspond with their distinct pathogenicity characteristics.

**Conclusions:**

Our findings suggest that the pneumococcus changes the short binding sites in the promoter regions of genes in a niche-specific manner to enhance its ability to disseminate from one host niche to another. This study provides a framework for an improved understanding of the dynamics of pneumococcal pathogenesis, and opens a new avenue into similar investigations in other pathogenic bacteria.

**Electronic supplementary material:**

The online version of this article (doi:10.1186/1471-2164-15-769) contains supplementary material, which is available to authorized users.

## Background

*Streptococcus pneumoniae* (the pneumococcus) continues to cause high morbidity and mortality worldwide, in spite of the availability of vaccines and antimicrobial therapies [1, 2]. Resistance of virulent *S. pneumoniae* to multiple antibiotics, particularly against beta-lactams is due to alterations in the structure of six penicillin-binding proteins, while macrolide resistance is mediated through the *erm(B)*, *mefA* or *mefE* genes [3]. This could partly be explained by the fact that the organism deploys an efficient virulence machinery during infection. Consequently, research efforts have been geared towards understanding the molecular mechanisms underlying the pathogenesis of pneumococcal disease [4–6]. However, due to technical difficulties associated with harvesting RNA for analysis of *in vivo* transcription patterns of pneumococci during pathogenesis, most studies have either studied the earlier stages of infection (nasopharynx and lungs) or used *in vitro* surrogates [7–10]. Recently, these challenges have been largely overcome by studies involving transcriptomic comparisons of differentially-regulated genes during penetration of deeper host tissues [11–13].

**Figure 1:**
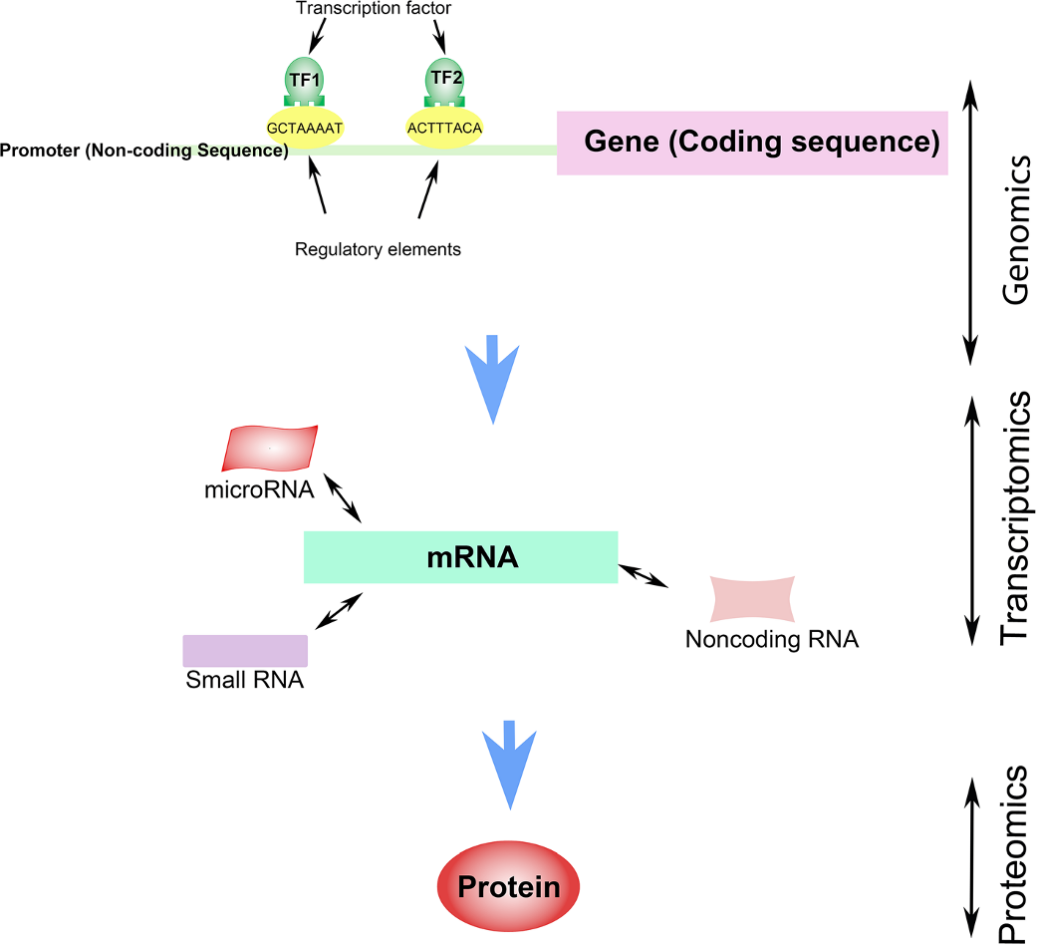
**Different layers of functional genomics and organism fitness, showing integration between upstream non-coding promoter region and downstream coding sequence leading to gene function.**

Notwithstanding these significant advances, progress on a comprehensive understanding of the dynamics of pneumococcal pathogenesis is still hampered by paucity of data on different levels of functional genomics (such as promoter activation and domain interaction), particularly for pneumococci with distinct pathogenicity characteristics. It is known that gene function is the outcome of harmony between the upstream non-coding promoter region and the downstream coding sequence [14] (Figure 1). Despite the prominent role of transcription factors (TFs) in controlling the expression of many genes [15, 16], their impact on pathogenesis of pneumococcal disease has not been studied in detail, probably due to their transient, and generally low expression levels. A study of 60 bacterial genomes showed that larger genomes harbour more TFs per gene than smaller ones [17]. This suggests that under complex conditions, gene expression, regulation and signal integration have been strongly selected to enable rapid adaptation to environmental conditions, triggering emergence of new strains. A differential fluorescence induction study showed that pneumococcal surface antigen (*psa)* promoter, which drives expression of the *psaBCA* operon involved in manganese uptake and virulence in *S. pneumoniae* [18, 19] is markedly activated during lung infection [20]. Recently, we found a novel transcriptional regulatory circuit consisting of two TFs (SmrC [SP_0927] and SP_0676) plays a significant role in pathogenesis and virulence of *S. pneumoniae* [21]. However, to date, there is no report that provides a detailed characterization of TFs (and their corresponding regulatory elements) and their activation patterns during pneumococcal translocation from the nasopharynx to deeper host tissues. The usual approach to address this problem involves the use of *in silico* analysis of promoter regions of differentially regulated genes and prediction of involved TFs, using the whole genomic sequence of a particular strain based on orthology [22–24]. Given the fact that functional specificity of proteins is conserved among orthologs [25], it is possible to compare whole genomes of *S. pneumoniae* with *Escherichia coli* to gain information on the TF and promoter activation map of pneumococcal virulence machinery.

The study of activated domains of expressed proteins in addition to promoter activation profiles can result in a better understanding of functional genomics. Compared to TFs, more information is available on pneumococcal pathogenesis at the level of expressed protein domains because of its use in vaccine design. The role of ATP-dependent transport and DNA binding domains in pneumococcal competence [26], histidine kinase in sensing environmental cues and virulence [27], and the role of PsaA in virulence [18, 28], are well documented. Genome wide analysis of histidine kinases and response regulators has led to the discovery of new response regulator loci [27], while mutation in the DNA gyrase domain has resulted in resistance to quinolones [29].

In the present study, we used functional genomics tools to examine the underlying molecular mechanisms that underpin the different pathogenicity characteristics of 3 pneumococcal strains at both promoter (transcriptomic) and domain (proteomic) activation levels. To our knowledge, this combined approach of characterizing *in vivo* microarray data has not been reported previously and can be used as a model for unraveling functional genomics attributes of other pathogenic bacteria.

## Results

For the purposes of this investigation, we have used 3 well-characterised pneumococcal strains: D39 (serotype 2), WCH43 (serotype 4) and WCH16 (serotype 6A). Mouse intranasal challenge experiments have shown that D39 and WCH43 are more virulent than WCH16. However, D39 causes severe pneumonia and high-grade bacteremia, while WCH16 and WCH43 have a propensity to translocate to the brain of infected mice [21, 30] (see Additional file 1: Figure S1). Nevertheless, WCH43 infection of mice demonstrates the “classical” disease progression from the nasopharynx to the lungs and dissemination to blood and then to the brain while WCH16 seems to progress directly to the brain with minimal lung and blood involvement [21, 31]. These striking differences in the pathogenicity and virulence characteristics of these strains make them ideal for comprehensive functional genomics analyses.

### Pneumococcal strains activate a variety of TFs during pathogenesis

Our initial bioinformatic analysis of microarray data for D39, WCH16 and WCH43 at 72 h post-infection of mice suggests that WCH16 up-regulates many genes in the lungs and brain, WCH43 up-regulates many genes in the brain, whereas D39 has significant genome activation in the blood. The list of up-regulated genes of D39, WCH16 and WCH43 during pathogenesis is presented in Additional files 2, 3, 4: Tables S1-S3.

We then characterized pneumococcal TFs capable of binding to a variety of up-regulated genes during pathogenesis and compared their activation profiles between D39, WCH16 and WCH43. Generally, the highest differential TF activation profile could be observed in the blood (see Additional file 5: Table S4). For D39, a total of 2196 transcription factor-binding sites (TFBs) or regulatory elements were found, while this number dramatically fell to 69 for WCH43 and just 11 for WCH16 in the blood. In this niche, TFs with the highest number (at least 100) of TFBs (such as *argR*, *codY*, *hup*, *rpoD*, *rr02*, *scrR* and *smrC*) were found in D39 (Table 1). The potential TFBs for each of the TFs are listed in Additional file 6: Table S5. The most active TF for all 3 strains in the blood was *rpoD*, although its activation rate is markedly different between the strains (3 predicted binding sites in WCH16, 26 in WCH43, and 767 in D39). Interestingly, a very high number of TFBs were associated with all these TFs (particularly *rpoD*, *hup* and *rr02*) in WCH16 and WCH43 during brain infection. We also compared activated TFs between D39, WCH16 and WCH43 during transition from the nasopharynx to the lungs. This revealed the same 7 activated TFs for WCH16 and WCH43, while 5 were activated for D39. Of these, *rpoD* was the most active TF for all 3 strains, with 74, 23 and 17 binding sites on the promoter regions of up-regulated genes in WCH16, WCH43 and D39, respectively (Table 1).

**Table 1:** **Transcription factors (TFs) with the highest number of binding sites on the promoter regions of up-regulated genes during pneumococcal pathogenesis**

TF	Lungs vs nasopharynx			Blood vs lungs			Brain vs blood	
	WCH16	WCH43	D39	WCH16	WCH43	D39	WCH16	WCH43
SP_0927 (*smrC*)	13	4			3	115	18	18
SP_1073 (*rpoD*)	74	23	17	3	26	767	94	113
SP_1113 (*hup*)	26	10	4	2	4	249	36	39
SP_1227 (*rr02*)	20	4	6	1	6	137	31	31
SP_1584 (*codY*)	19	3			8	175	28	25
SP_1725 (*scrR*)	16	4	1		1	72	15	17
SP_2077 (*argR*)	14	7	1	4	7	201	17	23

### Activated TFs with the highest number of TFBs cluster together

**Figure 2:**
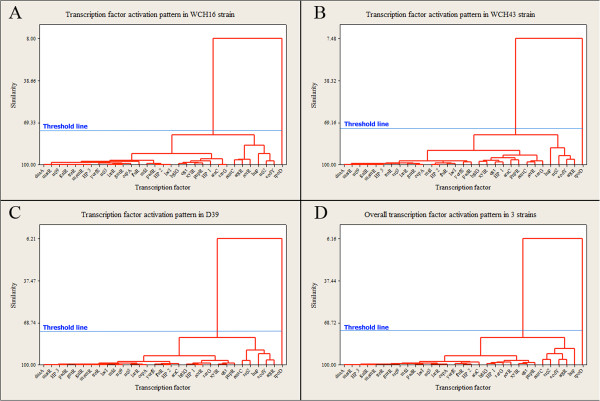
**Comparative transcription factor activation patterns in different** ***S. pneumoniae*** **strains.** Dendogram shows TF activation map for **(A)**, WCH16; **(B)**, WCH43; **(C)**, D39, and **(D)** for the 3 strains.

We sought to determine if there are any relationships or commonality between the activated TFs that we identified above in the 3 strains regarding their binding potential to the promoter regions of up-regulated genes during infection by hierarchical clustering analysis. Interestingly, we found that all activated TFs harbouring the highest number of TFBs (*argR*, *codY*, *hup*, *rr02*, *scrR* and *smrC*), except *rpoD*, clustered together with a high level of similarity (>70%) in all 3 strains (Figure 2, A-D). These results were further validated by clValid [32], using internal and stability cluster validation measures to design a threshold line to generate 2 clusters. The threshold line shows that *rpoD* has a distinct differential activation profile compared to the other TFs (Figure 2, A-D; see Additional file 7: Table S6).

**Figure 3:**
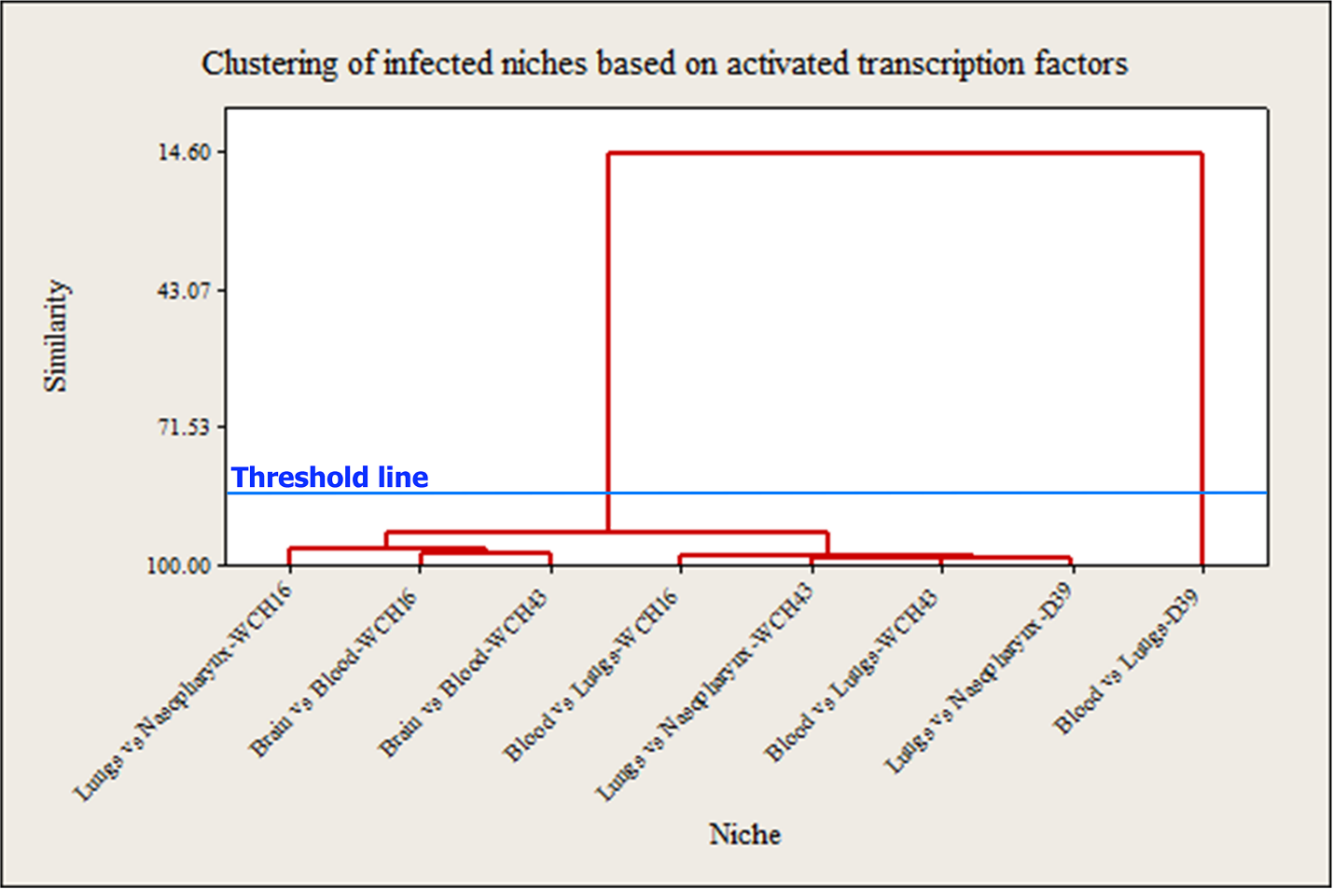
**Clustering profiles of activated transcription factors of** ***S. pneumoniae*** **strains across niches.**

We also compared the clustering profiles of all activated TFs across niches between the 3 strains. This showed that TFs that regulate the transition of WCH16 and WCH43 from the blood to the brain clustered together, with a very high level of similarity. Moreover, the TFs that regulate translocation from the nasopharynx to the lungs clustered with those activated during transition from the blood to brain for both strains, albeit at a lower level (Figure 3). We also found that TFs that control transition from the nasopharynx to the lungs for D39, and those responsible for transition from the lungs to blood for WCH43 clustered together, while the TFs that regulated transition from lungs to blood in D39 were quite distinct. These clustering patterns were also validated by c1Valid, and based on the 2 generated clusters after applying threshold line, blood *vs* lungs in strain D39 shows a distinct profile of activated TFs (Figure 3, Additional file 7: Table S6). These differences in TF activation profiles across niches are consistent with the bioluminescence patterns of the 3 strains in mice (Additional file 1: Figure S1).

### Activated TFs exhibit different behavior between pneumococcal strains

We used discriminant function analysis to compare the relative impact (weight) of TFs between the 3 strains in various niches. Comparison of D39 and WCH16 showed that both strains have relatively similar discriminant models in the lungs. In the blood, a large number of TFs have opposite signs between D39 and WCH16. This highlights differential regulatory backgrounds of both strains in the blood (see Additional file 8: Table S7). However, the discriminant modelling algorithm indicate that D39 and WCH43 have similar regulatory backgrounds in the lungs but are different in the blood. The discriminant models of WCH16 and WCH43 in the lungs and brain show that the activated TFs have similar coefficient values and signs in both strains (Additional file 8: Table S7). Interestingly, these strains choose different models during progression from lungs to blood. In this instance, of the 7 highly activated TFs, *codY* was the most significant predictor discriminating WCH16 from WCH43 in the blood, where its coefficient was -38 in WCH16, compared to +13 in WCH43.

Given the predicted discriminant function for *codY* between these 3 strains, we hypothesized that a mutant of *codY* will show distinct pathogenic profiles between these strains *in vivo*. However, we noted that a previous manuscript reported that *codY* inactivation in D39 is difficult due to its essentiality [33] and suggested further work in other clinical strains to establish if this is a general feature of pneumococci. Therefore, we attempted to delete *codY* in D39, WCH16 and WCH43 by targeted mutagenesis using the overlap extension strategy, as described in Methods. Surprisingly, we obtained putative mutants in all 3 strains, which were verified to be correct by PCR and sequencing using primers flanking the *codY* ORF. We examined if the putative *codY* mutants carried the permissive amino acid (Q_166_ → Stop) mutation in FatC (SP_1870) and/or amino acids (D_480_ → Y; D_487_ → Y) and amino acids 1–81 deletion mutations in AmiC (SP_1890) reported previously [33]. This was carried out by PCR and sequencing of the *amiC* and *fatC* genes of 2 independent mutants from each of the 3 strains. However, we could not detect any of these reported changes. Next, we tested the fitness of each of these putative *codY* mutants in an *in vitro* competition with the isogenic wild type over 4.5 hrs. Surprisingly, we found that the mutant of all 3 strains was completely out-competed by the wild type. Output ratios were 1:1300, 1:655 and 1:1900, for D39Δ*codY*, WCH16Δ*codY* and WCH43Δ*codY* mutants, respectively at 3 h. We concluded that while the *codY* mutation might have been tolerated in these strains, the mutation is unstable, in agreement with the finding of Caymaris and colleagues regarding the essentiality of CodY [33]. We also found that during *in vitro* growth, the antibiotic resistance selection marker in the *codY* mutant strains is lost in the absence of selection. This was verified by replica-plating cells grown on antibiotic plates onto plates with or without antibiotic; the relative rate of loss of selection was more rapid in the WCH16Δ*codY* (1:6.5), compared to 1:5, and 1:1.4, for the WCH43Δ*codY* and D39Δ*codY* putative mutants, respectively.

**Figure 4:**
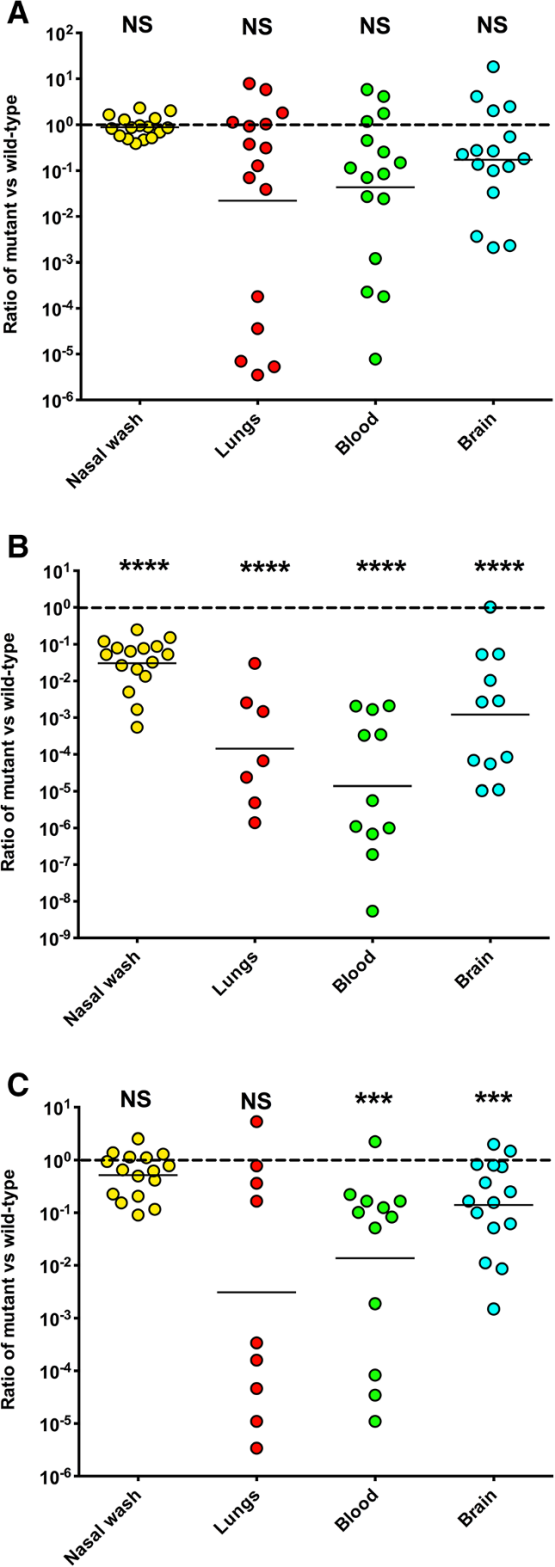
**Competition experiments between wild type D39 (A), WCH16 (B), WCH43 (C) and their** ***codY*** ^**ind**^ **isogenic counterparts in the nasopharynx, lungs, blood and brain (n = 16) 36 h post-i.n. infection with equal numbers (approx. 2.5 × 10** ^**6**^ **CFU) each of wild type and isogenic derivative.** Datum points are the ratio of *codY* ^ind^ bacteria to wild type for each animal. The horizontal broken line represents a 1:1 ratio. The horizontal solid line denotes the geometric mean ratio for each comparison (NS = Not Significant; *** *P* < 0.001; **** *P* < 0.0001; one sample *t*-test, two-tailed).

As a consequence of these findings, we explored an alternative approach to investigating the discriminant function of CodY in the 3 strains. We placed *codY* under the expression of *ami* promoter and transformed this into the 3 strains, generating D39[*codY*]^ind^, WCH16[*codY*]^ind^, and D39[*codY*]^ind^, respectively. Next, we tested the fitness of these *codY*^ind^ strains in an *in vitro* competition experiment with their otherwise isogenic wild-type derivatives over 4.5 hrs. In this model, there was no difference in bacterial counts of the *codY*^ind^ strains compared to their isogenic wild-type counterparts at any of the time points tested (not shown). We then investigated the effect of over-expressing CodY on the *in vivo* fitness of the *codY*^ind^ strains relative to their respective wild type counterparts in a mouse intranasal (i.n.) competition experiment over a 36 h period, as described in Methods. Our analysis shows that there was no significant difference in the ability of the D39[*codY*]^ind^ strain to colonise the nasopharynx or invade the lungs, blood or brain (Figure 4A). However, the WCH16[*codY*]^ind^ strain was massively out-competed by its wild-type counterpart in all these niches (Figure 4B). Interestingly, the WCH43[*codY*]^ind^ strain was only out-competed by the isogenic wild-type strain in its ability to invade the bloodstream and brain (Figure 4C). Together, these analyses confirm the bioinformatics prediction of the discriminant function of CodY in WCH16, consistent with its role in controlling the expression of many genes involved in pneumococcal metabolism and virulence [33, 34].

### Up-regulated genes are predicted to be co-regulated by similar sets of TFs in various niches

**Figure 5:**
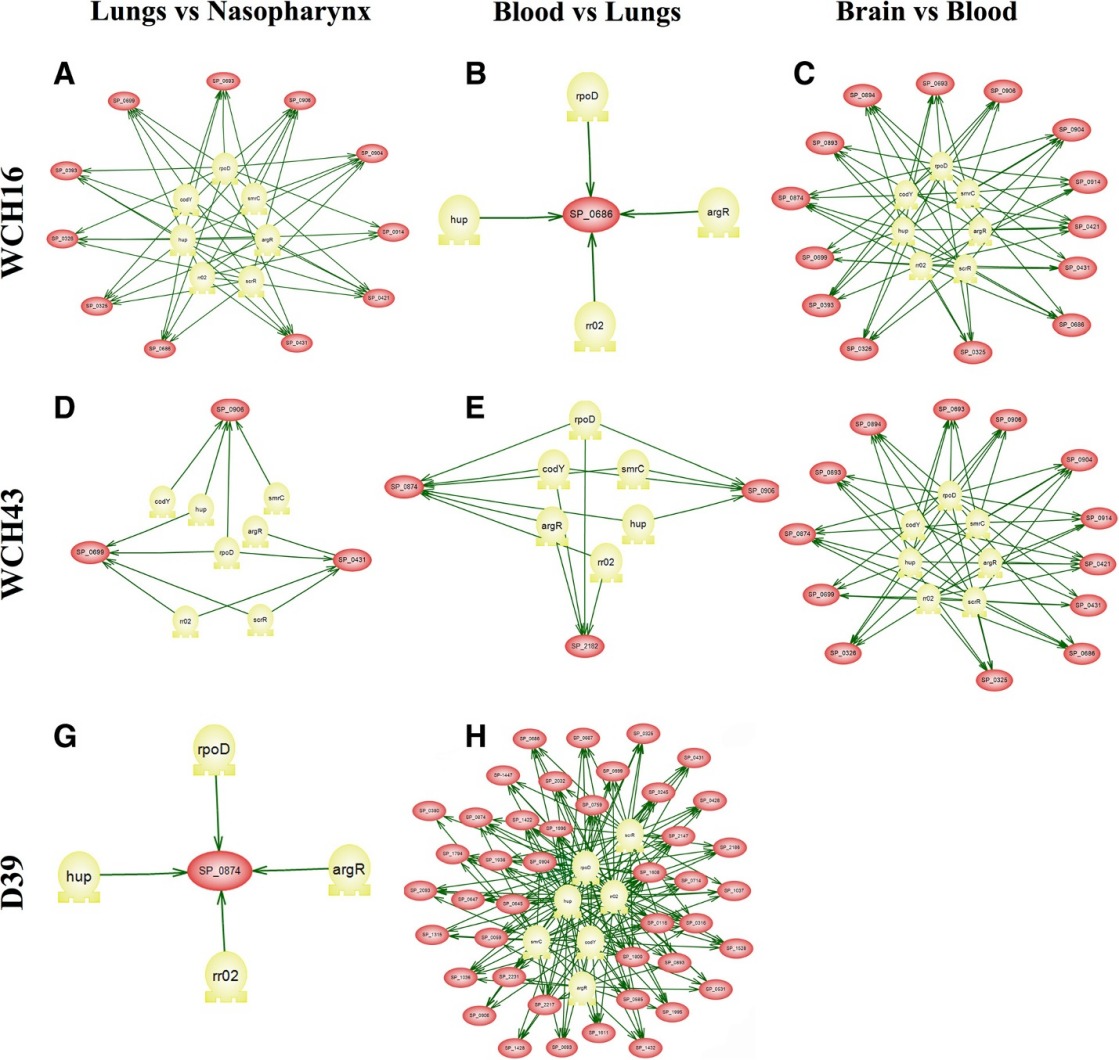
**Transcription factor-based regulatory network analysis of** ***S. pneumoniae*** **strains during infection. (A)** Regulatory network underlying transition of WCH16 from Nasopharynx to Lungs. **(B)** Regulatory network underlying transition of WCH16 from Lungs to Blood. **(C)** Regulatory network underlying transition of WCH16 from Blood to Brain. **(D)** Regulatory network underlying transition of WCH43 from Nasopharynx to Lungs. **(E)** Regulatory network underlying transition of WCH43 from Lungs to Blood. **(F)** Regulatory network underlying transition of WCH43 from Blood to Brain. **(G)** Regulatory network underlying transition of D39 from Nasopharynx to Lungs. **(H)** Regulatory network underlying transition of D39 from Lungs to Blood.

We then attempted to identify genes (with at least 4 TFBs in their promoter regions) that are co-regulated by the same 7 highly activated TFs in the lungs, blood or brain. This allowed us to construct a series of TF activation networks for the 3 strains (Figure 5, A-H; see Additional file 9: Table S8). Overall, our results show that the same groups of genes were co-regulated by similar sets of TFs in the lungs and brains of mice infected with WCH16, and in the brains of mice infected with WCH43. These results correlate with the TF activation profiles observed for these strains in those niches (as shown in Figure 3). Interestingly, only SP_1647 (a metallo-endopeptidase; in the lungs of D39-infected mice), and SP_1058 (hypothetical protein; in the blood of WCH16-infected mice) were found to be potentially co-regulated by *argR*, *hup*, *rpoD* and *rr02*. We also observed that D39 showed a distinct set and high number of up-regulated genes in the blood. The majority of these genes are co-regulated by 4 TFs, except for a set of genes involved in the pentose phosphate pathway (SP_0316-SP_0320) that are co-regulated by all but one (*smrC*) of the 7 highly activated TFs (Figure 5H; Additional file 9: Table S8). A view of the genome maps of sequenced *S. pneumoniae* strains suggests these genes are grouped in transcriptional units, although the sequence of SP_0316 is very short (108 bp) and is absent in most of these strains (except *S. pneumoniae* TIGR4 and *S. pneumoniae* TCH8431/19A). Search for orthologous genes and clustering analysis also shows a high level of identity and conservation for SP_0317-SP_0320 in other streptococci (such as *S. agalactiae*, *S. faecalis*, *S. faecium* and *S. pyogenes*) and in *Bacillus subtilis*. These results are reminiscent of redundancy of TF–TFB interactions as described for overlapping regulons like SoxS, MarA and Rob of *E. coli* [35], and among PrfA/CtsR/HrcA and alternative sigma factors (σ^B^, σ^C^, σ^H^, and σ^L^) of *Listeria monocytogenes* [36] and among the ς^X^ and ς^W^ regulon in *B. subtilis* [37] in which different sites are able to recruit the same TF, while different TFs can recognize similar sites.

Our analysis also included the identification of genes that are commonly up-regulated by each of the 7 highly activated TFs in the lungs, blood or brain for the 3 strains. We restricted our analysis to include the genes that harbor a minimum of 4 TFBs in their promoter regions in at least 2 of the 3 strains. This showed that the same set of genes (except SP_0698) are commonly up-regulated in the lungs and brain for WCH16 and WCH43 (see Additional file 10: Table S9). However, these genes were not detected during lung infection by D39. By contrast, only one gene (SP_1329), a putative N-acetylneuraminate lyase, was up-regulated in WCH43 and D39 in the blood. These results are complementary to data presented in Additional file 8: Table S7.

### Genes harboring higher numbers of regulatory elements constitute a novel index of quality-based gene selection

We developed a new quality-based selection index based on mining of the number of regulatory elements on the promoter regions of up-regulated genes controlled by the 7 highly activated TFs. Specifically, we hypothesized that genes containing higher numbers of TFBs are likely to play key roles in pneumococcal pathogenesis because of their ability to host more TFs. Of the up-regulated genes during infection of lungs and brain by WCH16 and WCH43, SP_0698 (ABC-2 type transport system permease protein) was predicted to contain the highest number of TFBs (13) in its promoter region (see Additional file 11: Table S10). In the blood, WCH43 up-regulates SP_2182 (a bacteriocin_IIc-type protein) with 18 TFBs in its promoter region. However, in D39, SP_0124 and SP_0125 (bacteriocin_IIc-type proteins), SP_0874 and SP_1036 (hypothetical proteins), and SP_1608 (a DadA-family oxidoreductase) (with 23, 26, 25, and 13, TFBs, respectively) were up-regulated.

GO analysis of up-regulated genes containing higher number of TFBs in lungs *vs* nasopharynx suggests that these genes are involved in key metabolic processes such as NADPH activity, fatty acid biosynthetic process, sugar phosphotransferase system, membrane development, NAD binding, and proteolysis. These processes are important during colonization of the nasopharynx and translocation to lungs by *S. pneumoniae*. In the blood vs lungs comparisons, genes containing high number of TFBs enrich GO processes of ion binding (magnesium ion binding/iron ion binding), transporter activity, metabolic process, as well as ion transmembrane transport. These processes are vital for survival of *S. pneumoniae* in the blood, highlighting the important roles of such up-regulated genes with high TFBs in their promoter regions in the blood. GO analysis of up-regulated genes with high TFBs in the brain *vs* blood comparison were found to be involved in important processes such as NADPH activity, sugar:hydrogen symporter activity, glutamine biosynthetic process, fatty acid biosynthetic process, sugar phosphotransferase activity, kinase activity, carbohydrate transmembrane transport, ion transmembrane transport, and NAD binding. These processes facilitate the translocation, survival and adaptation of WCH16 and WCH43 in the brain.

### Domain activation profiles of different pneumococcal strains during pathogenesis

We reasoned that the study of activated domains of expressed proteins in addition to promoter activation profiles could result in a better understanding of functional genomics because of its use in vaccine design. Therefore, we predicted the complete domain activation catalogue of WCH16, WCH43 and D39 during infection of various host tissues. Accordingly, WCH16 (which is less virulent than WCH43 and D39) is predicted to have the highest amount of its domain activation in the lungs, with 74 predicted domains, compared to 31 for D39 and 23 for WCH43 (Table 2 and Additional file 12: Table S11). Our analysis also suggests that WCH16 has relatively low activation in the blood, with only one predicted activated domain [(N6_N4_Mtase (DNA methyltransferase)]. In comparison, WCH43 is predicted to activate 14 domains in the blood, while D39 is predicted to exhibit the most remarkable profile, activating 470 possible domains in the blood. It is also predicted that energy producing domains such as ATPase family associated, ATP-synt_ab_N (ATP synthase alpha/beta subunits) and ABC transporters are used by D39 to maintain fulminant bacteremia (Additional file 12: Table S11).

**Table 2:** **Number of activated protein domains by virulence machinery of different** ***S. pneumoniae*** **strains during movement from the nasopharynx → lungs → blood → brain**

Strain	Lungs vs nasopharynx	Blood vs lungs	Brain vs blood
WCH16	74	1	105
WCH43	23	14	110
D39	31	470	0

Comparison of the activated domain profiles of WCH16 and WCH43 in the brain revealed that the number of activated domains for both strains are essentially similar (105 and 110, respectively; Table 2). The only slight difference is that in WCH43 more activated domains including energy-producing (such as ATP-grasp), two-component regulatory system, tRNA, ribosomal and PTS systems domains were found.

## Discussion

In this work, we have used a functional genomics-based approach to provide a comprehensive prediction of TF activation profiles and domain expression patterns of 3 different *S. pneumoniae* strains during pathogenesis. It has been suggested that the TF and promoter activation map of an organism can be obtained by comparison with the fully annotated map of an ortholog [22–24] given the fact that functional specificity of proteins is conserved among orthologs [25]. Therefore, we exploited the available *E. coli* transcriptional regulation data to gain information on the orthologous TF and promoter activation map of 3 *S. pneumoniae* strains (WCH16, WCH43 and D39) during pathogenesis. However, it has been argued that such methods have shortcomings when applied to distantly related organisms [38]. We attempted to address this potential drawback by using an approach that predicts the function of genes based on the similarity/difference in the pattern of TFBs. The identified potential TFBs and their organization modules provide another way to understand gene expression and regulation during pathogenesis. Taking into account these approaches, we present a strategy for selection of novel antibacterial targets and vaccine candidates.

In a recent investigation [21], we introduced the concept of bacterial niche-specific virulence gene expression during pathogenesis by qualitative comparisons of transcriptomic data of one niche versus the previous niche. In the present study, we carried out an in-depth analysis of the TF-regulatory network that govern this transition in order to shed light on the virulence factors that *S. pneumoniae* employs to breach host tissue barriers. Our analysis showed that 7 TFs (*argR*, *codY*, *hup*, *rpoD*, *rr02*, *scrR* and *smrC*) were highly activated in all 3 strains *in vivo* and were capable of binding to a large number of up-regulated genes. We suggest that these TFs might constitute the regulatory backbone that underpins pneumococcal pathogenesis. We also found that these TFs were used by D39 in blood and by WCH16 and WCH43 during brain infection.

A close examination of activated TFs of the 3 strains suggests that *S. pneumoniae* adapts to each host niche during infection by increasing the number of TFs deployed (and their TFBs). As a corollary, the number of activated TFs is reduced if the bacteria are unable to adapt to a particular niche (e.g. WCH16 in the blood). Not surprisingly, *rpoD* was the most active TF for all 3 strains in all niches, consistent with its role in promoting the attachment of RNA polymerase to specific initiation sites during transcription. The observed similarity between the predicted TF activation profiles and their clustering patterns between distinct niches suggests that different *S. pneumoniae* strains might activate the same set of TFs and regulatory elements at different sites of infection. This is further supported by discriminant function analysis of the TFs in various niches, which highlights differential regulatory backgrounds of the 3 strains in the blood. We also provide experimental evidence that validates the predicted discriminant function for CodY between the 3 strains by showing distinct pathogenic profiles of their otherwise isogenic *codY* induced derivatives in various anatomical niches of mice. The results are consistent with our *in vivo* microarray and real-time RT-PCR data showing differential expression of CodY-regulated virulence genes such as *aliA*, *ilvH* and *piuA* in various niches of mice [13], and are also in agreement with results from other workers [33]. Furthermore, the predicted domain activation catalogues of these strains during pathogenesis suggests that their functional domain profiles differed mainly in the blood, with only one domain (DNA methyltransferase) activated for WCH16, 14 for WCH43 and 470 domains for D39. These results are consistent with gene ontology classifications for WCH16 and WCH43 [21, 39], and in agreement with our published observations showing that these 3 strains display distinct pathogenicity characteristics [21, 31, 39].

Our analyses also showed that some genes could potentially be regulated by multiple TFs. For example, SP_1647 (a metallo-endopeptidase) was predicted to be regulated by *argR*, *hup*, *rpoD* and *rr02* in the lungs of D39-infected mice. Notably, this gene has recently been characterized to have a role in pneumococcal attachment and internalization in host epithelial (A549) and endothelial (HUVEC) cell lines [40]. We also developed a new quality-based strategy for selection of genes important for pathogenesis, based on mining of the number of TFBs in their promoter regions. GO analysis of such genes allowed us to propose that number of TFBs in the promoter regions of up-regulated genes is an important and novel index for selection of genes that play key roles at distinct stages of pneumococcal pathogenesis. In this context, we found, amongst other genes, a group of class IIc bacteriocins that harbor the highest number of TFBs in WCH43 and D39 harvested from the blood. Bacteriocins have been shown to be involved in fratricide in *S. pneumoniae*, a phenomenon that favors natural competence and pathogenesis in this organism [41, 42]. Additionally, a putative N-acetylneuraminate lyase (SP_1329) was up-regulated by both WCH43 and D39 in blood, suggesting a need for this enzyme for metabolic adaptation of these strains in this niche.

It has been suggested that regulation of pneumococcal virulence proteins is very complex and multifactorial, and likely involves overlapping regulatory mechanisms [43]. We found that all the highly activated TFs *argR*, *codY*, *hup*, *rr02*, *scrR* and *smrC*), except *rpoD*, clustered together in all 3 strains. This is a significant finding, implying redundancy in the regulatory roles of these TFs during infection, as the functions of these TFs in pathogenesis and regulation of virulence gene expression have hitherto been studied in isolation [7, 33, 34, 44–47]. Indeed, this notion is exemplified by our recent work, which showed that a defined mutation of *smrC* in a WCH43 background did not completely abrogate virulence [21]. In the same context, we showed in Figure 4 that the same groups of up-regulated genes are predicted to be co-regulated by similar sets of TFs. These findings might explain a fundamental problem associated with prevention and control of pneumococcal disease, as it appears that the task of identifying the principal targets of intervention would be challenging. Therefore, a thorough analysis of the important regulatory pathways employed by *S. pneumoniae* in different *in vivo* niches would likely improve our knowledge of pneumococcal pathogenesis and identify novel targets for intervention.

## Conclusions

Our findings suggest a possible evolutionary strategy for the pneumococcus in which the evolution occurs in non-coding promoter regions of genes during infection. Specifically, the pneumococcus changes the short binding sites in the promoter regions of genes instead of alteration of coding sequences in a niche-specific manner to generate more virulent strains. There are two important caveats of this study. Firstly, we utilized the fully annotated *E. coli* promoter listings to pull out the orthologous TFs in *S. pneumoniae*, which might not represent the full array of active promoters in the pneumococcus. Secondly, the microarray slides used in this study cannot detect the expression of unique WCH16-specific genes that are absent in TIGR4 and R6 genomes represented on the slides. Nevertheless, this study is the first such investigation; it provides a framework towards a better understanding of the dynamics of pneumococcal pathogenesis, and opens new avenues into similar investigations in other pathogenic bacteria.

## Methods

### Bacterial strains and growth conditions

The pneumococcal strains used in this study were D39 (serotype 2; Sequence Type 595), clinical blood isolates WCH43 (serotype 4; Sequence Type 205) and WCH16 (serotype 6A; Sequence Type 4966). Previous mouse intranasal challenge experiments in our laboratory with these strains indicated that D39 causes fulminant bacteraemia; WCH43 demonstrates the “classical” disease progression from the nasopharynx to the lungs, followed by dissemination to the blood and then to the brain, while WCH16 demonstrates minimal lung and blood involvement before translocation to the brain [31] Serotype-specific capsule production was confirmed by Quellung reaction, as described previously [48]. Opaque-phase variants of the strains, selected on Todd-Hewitt broth supplemented with 1% yeast extract (THY)-catalase plates [49], were used in all animal experiments. Before infection, the bacteria were grown statically at 37°C in serum broth (SB) to *A*_600_ of 0.16 (equivalent to approx. 5 × 10^7^ CFU/ml).

### Microarray analysis of RNA extracted from different mouse tissues during pathogenesis

For this investigation, we analyzed 72 h-microarray data of *in vivo*-derived RNA samples obtained from our previous studies with WCH16 and WCH43 [12, 13] as well as that generated for D39 [12, 13, 31, 50]. Our previous studies have also established that after intranasal infection of CD-1 mice, the number of pneumococci recovered from various tissues peaked at 72 hr after which the mice succumbed to infection quite rapidly [31]. Therefore, we focused on this time point for further differential gene expression analyses. This was carried out by a modified two-color microarray analysis where the relative expression of each gene in one niche was calculated in comparison to expression in the previous niche when bacteria moves from the nasopharynx to lungs to blood and then to brain. Differential gene expression values with *p* < 0.05 were considered as statistically significant (one-sample *t*-test). In the case of multiple comparisons, T- Hotelling using Minitab 16 package (http://www.minitab.com) was used to adjust *p*-values and decrease type I error. The goal of this analysis was to unravel pneumococcal gene expression patterns in the nasopharynx, lungs, blood and brain of mice.

### Promoter extraction of up-regulated genes

**Figure 6:**
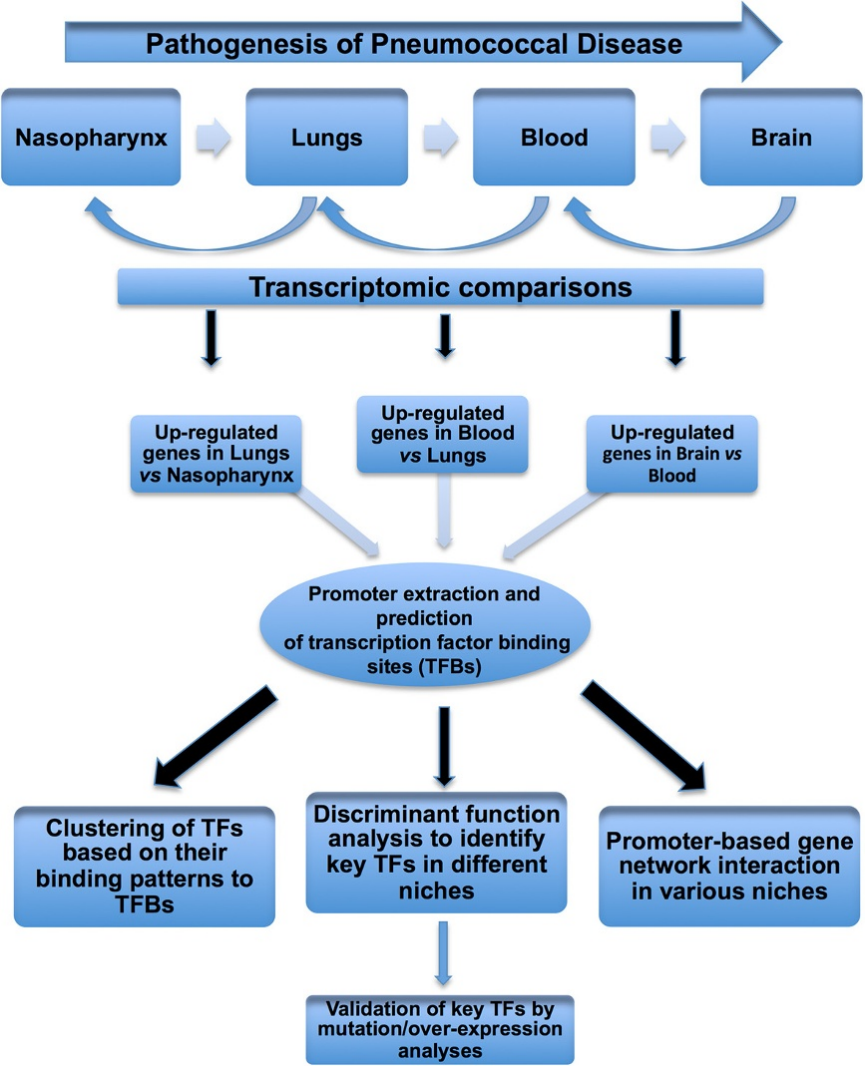
**Schematic representation of the step-by-step procedure for extraction of promoter regions up-regulated genes of** ***S. pneumoniae*** **during infection for TF discovery, clustering, discriminant function analyses, promoter-based gene network interaction and mutational studies.**

In order to carry out a detailed characterization of the pneumococcal TFs that are active in each host niche during infection, a series of analyses were performed (Figure 6). Firstly, pneumococcal genes that were significantly up-regulated in each niche at 72 h were identified by pairwise comparison of expression levels of each gene in one niche versus the previous niche (e.g. lungs *vs* nasopharynx, blood *vs* lungs, brain *vs* blood). Databases containing full genomic sequences of *S. pneumoniae* TIGR4 and D39, such as Microbesonline (http://www.microbesonline.org/) [51, 52] and KEGG (http://www.genome.jp/kegg/), were used to find the genomic location of up-regulated genes. Then, the potential promoter regions of up-regulated genes in different niches for all 3 *S. pneumoniae* strains were extracted as follows. The upstream region between each up-regulated gene (or operon) and the next gene (or operon) was extracted from KEGG, Microbesonline database, or Genome-Tools Web Interface (http://genome-tools.sourceforge.net/cgi-bin/genome-tools-web-interface.pl) [53]. The promoter analysis covers the entire promoter region of each gene or operon. Existence of a promoter and its -10 and -35 sites was confirmed for up-regulated genes using BPROM algorithm (http://linux1.softberry.com/berry.phtml?topic=bprom&group=programs&subgroup=gfindb). The algorithm predicts potential transcription start positions of bacterial genes regulated by sigma70 promoters using linear discriminant function. As the prediction tools for determining TFBs are not as complete in *S. pneumoniae*, we used the TFB prediction tool of *E. coli* to predict TFBs on the extracted promoter regions for each gene by pattern matching using the BPROM tool at the SoftBerry portal (http://linux1.softberry.com/berry.phtml) [54]. TFs were predicted for all extracted promoter sequences of up-regulated genes in all niches of WCH16, WCH43, and D39 using RegPrecise (http://regprecise.lbl.gov/RegPrecise/) [55] and BPROM web applications. The number of regulatory elements (TFBs) in the promoter region of each gene was recorded. In each niche, TFs with the highest number of TFBs in the promoter regions of up-regulated genes were determined for each strain. The number of TFBs for each TF was also recorded. We chose 7 TFs with at least 100 TFBs in their promoter regions as a cut-off for further analysis.

### Comparative analysis of TF activation in different niches

Based on promoter analysis of up-regulated genes, a map of TF activation was estimated in different niches for the 3 strains. To obtain a clear understanding of similarities and/or differences in TF activation, TFs were clustered according to the number of TFBs on promoter regions of up-regulated genes during infection by hierarchical clustering analysis (HCA). This was achieved by count data (number of possible binding sites of each TF to the promoter regions of up-regulated genes in each niche comparison) for all strains. When a particular TF does not have a potential binding site on promoter regions of up-regulated genes, a value of “0” was placed in the data. For each strain, similarities/differences in different TF activation patterns (in terms of potential binding to promoter regions of up-regulated genes) were calculated by average linkage method based on Euclidean distance measure [56]. Dendograms were designed based on calculated similarity matrixes by Minitab 16 package (http://www.minitab.com) to visualise differential binding patterns. In addition, clustering of overall TF activation map in all strains and niches was carried out as described above. The hierarchical clustering analyses were validated using c1Valid R Package [32], by internal and stability cluster validation measures. Internal measures represent the compactness, connectedness, and separation of the cluster partitions by Connectivity, Silhouette Width and Dunn Index, where lower Connectivity but higher Dunn and Silhouette represent better scores. Stability measures assess the results from the full clustering data based on removing each column, one at a time. The Stability measures evaluated were: Average proportion of non-overlap (APN), Average distance (AD), Average distance between means (ADM) and Figure of merit (FOM); lower APN, AD, ADM, and FOM represent better scores. We then specified best threshold line to generate 2 clusters.

### Discriminant function analysis

We used linear discriminant statistical analysis to determine the functions of TFs and used this information to discriminate between the impact of a particular TF in each niche between two groups (e.g. WCH16 vs WCH43, WCH16 vs D39, WCH43 vs D39). This predictive algorithm provides an accurate comparative index of TF function, whereby TFs with relative higher coefficient values (either positive or negative) have significant influence/impact in a particular niche between the two groups being compared.

### GO analysis of up-regulated genes with high number of TFBs in their promoter regions

Up-regulated genes containing higher number of TFBs during pneumococcal pathogenesis were subjected to GO classification for their functional association categories with respect to molecular function and biological process, using our recently developed bacterial GO web application [39, 57].

### TF–based network construction of pneumococcal pathogenesis

A database was made based on the results of promoter analysis linking the genes to TFs. TF-based regulatory networks were designed in different niches for each strain based on the developed database. The database relationships were visualised using PathwayStudio 9 (Elsevier, USA). The database is available upon request.

### Extraction of domain profile of up-regulated genes

Protein sequences of up-regulated genes of WCH16, WCH43, and D39 were extracted from PIR (Protein Information Resource) databases (http://pir.georgetown.edu/cgi-bin/batch.pl). Then, domains of each protein sequence were extracted from pfam database (http://pfam.sanger.ac.uk/) and CLC Genomics Workbench 6 (http://www.clcbio.com/). For domain prediction, E-value = 0.0000001was used to extract the significant domains.

### Construction of mutants and assessment of bacterial growth

*S. pneumoniae* derivatives with marked mutations in *codY* were constructed in the 3 strains. Mutants were constructed by overlap extension PCR [58, 59] and validated by PCR and sequencing to be in-frame deletion mutation replacements. All PCR procedures were performed with the Phusion High Fidelity Kit (FINNZYMES). The primer pairs used for construction and validation of the mutants are listed in Additional file 13: Table S12. A recombinant plasmid over-expressing *codY* was constructed by driving its expression from *ami* promoter using pAL2 plasmid [60] in which the *luxABCDE* cassette had been deleted (pAL2Δ*luxABCDE*). The recombinant construct was transformed into the 3 strains, generating D39[*codY*]^ind^, WCH16[*codY*]^ind^, and D39[*codY*]^ind^, respectively. Induction of *codY* in these strains was confirmed by real-time RT-PCR analysis.

*In vitro* competition experiments were performed as described previously [21]. In this assay, mutant (or *codY*^ind^) and wild-type bacteria were grown to *A*_600_ in SB and then mixed at an input ratio of 1:1 in SB. At 1.5 and 3 h post incubation, an aliquot of each sample was serially diluted in SB and plated on blood agar and blood agar with a selective antibiotic to determine the ratio of mutant (or *codY*^ind^) to wild-type bacteria. Each competition experiment was repeated at least twice. Competitive indices were calculated as the ratio (±SEM) of mutant (or *codY*^ind^) to wild-type bacteria recovered at each time point adjusted by the input ratio.

### Mixed infection experiments

Competition experiments were carried out essentially as described previously [12]. Briefly, 8 mice were anesthetized by intraperitoneal injection of pentobarbital sodium (Nembutal; Rhone-Merieux) at a dose of 66 mg per g of body weight and separately challenged i.n. with 50 μl suspension containing approx. 2.5 × 10^6^ CFU of either wild-type or the isogenic *codY*^ind^ strain. At 36 h post-challenge, mice from each separate infection experiment were sacrificed, bacteria were enumerated from the nasopharynx, lungs blood and brain, by plating on blood agar and blood agar with a selective antibiotic to determine the ratio of the *codY*^ind^ strain to wild-type bacteria. Each competition experiment was repeated at least twice. Competitive indices were calculated as the ratio (±SEM) of *codY*^ind^ strain to wild-type bacteria recovered in each niche adjusted by the input ratio.

### Ethical considerations

Outbred 5- to 6-week-old female CD1 (Swiss) mice were used in all experiments. The Animal Ethics Committee of The University of Adelaide approved all animal experiments (Project Number: S-2013-053). The study was conducted in compliance with the Australian Code of Practice for the Care and Use of Animals for Scientific Purposes (7th Edition 2004) and the South Australian Animal Welfare Act 1985.

### Data access

The data reported in this paper are archived at the following databases: BμG@Sbase (http://bugs.sgul.ac.uk/E-BUGS-130 and http://bugs.sgul.ac.uk/E-BUGS-133, and also ArrayExpress (accession number E-BUGS-130, and E-BUGS-133).

## Authors’ information

James C Paton, Abiodun D Ogunniyi and Esmaeil Ebrahimie Joint Senior authors.

## Electronic supplementary material

Additional file 1: Figure S1: Bioluminescent imaging of mice infected with WCH16, WCH43 or D39 at 72 h post-challenge, showing bacteria in the nasopharynx, lungs, blood and brain, for WCH16 and WCH43, and in the nasopharynx, lungs and blood for D39. (PDF 513 KB)

Additional file 2: Table S1: List of up-regulated genes of *S. pneumoniae* WCH16 during pathogenesis. (DOCX 163 KB)

Additional file 3: Table S2: List of up-regulated genes of *S. pneumoniae* WCH43 during pathogenesis. (DOCX 168 KB)

Additional file 4: Table S3: List of up-regulated genes of *S. pneumoniae* D39 during pathogenesis. (DOCX 155 KB)

Additional file 5: Table S4: Transcription factor (TF) activation catalogue of 3 pneumococcal strains with different pathogenic profiles during infection. (DOCX 125 KB)

Additional file 6: Table S5.: List of highly activated transcription factors (TFs) during pathogenesis of *S. pneumoniae* WCH16, WCH43, and D39 and their potential regulatory elements. (DOCX 86 KB)

Additional file 7: Table S6: Validation of TF activation maps for *S. pneumoniae* WCH16, WCH43 and D39, and for all 3 strains and validation of the clustering profiles of activated TFs of all 3 *S. pneumoniae* strains across niches (Figures 2A, 2B, 2C, 2D and Figure 3). (DOCX 32 KB)

Additional file 8: Table S7: Comparative discriminant function analysis of *Streptococcus pneumoniae* transcription factors during pathogenesis. (DOCX 52 KB)

Additional file 9: Table S8: Genes co-regulated by the same Transcription factors (TFs) during pneumococcal pathogenesis [SP_0927 (*smrC*), SP_1073 (*rpoD*), SP_1113 (*hup*), SP_1227 (*rr02*), SP_1584 (*codY*), SP_1725 (*scrR*), and SP_2077 (*argR*)]. (DOCX 152 KB)

Additional file 10: Table S9: Transcription factor-specific upregulated genes shared by *S pnemoniae* WCH16, WCH43 and D39 during pathogenesis. (DOCX 16 KB)

Additional file 11: Table S10: *S. pneumoniae* up-regulated genes under the control of highly activated transcription factors and the number of transcription factor binding sites (TFBs) in their promoter regions. (DOCX 144 KB)

Additional file 12: Table S11: Domain activation profiles of *S. pneumoniae* WCH16, WCH43, and D39 during transition from the nasopharynx → lungs → blood → brain. (DOCX 158 KB)

Additional file 13: Table S12: Primers for construction of mutants, cloning, sequencing and real-time RT-PCR analysis. (DOCX 85 KB)
