# Supplementary material for: A functional genomics catalogue of activated transcription factors during pathogenesis of pneumococcal disease
Source: BMC Genomics. 2014 Sep 8;15(1):769. doi: 10.1186/1471-2164-15-769 (PMC4171566; doi:10.1186/1471-2164-15-769)
Supplement: Supplementary file 1 — Additional file 1: Figure S1: Bioluminescent imaging of mice infected with WCH16, WCH43 or D39 at 72 h post-challenge, showing bacteria in the nasopharynx, lungs, blood and brain, for WCH16 and WCH43, and in the nasopharynx, lungs and blood for D39. (PDF 513 KB) [file 12864_2014_6462_MOESM1_ESM.pdf]

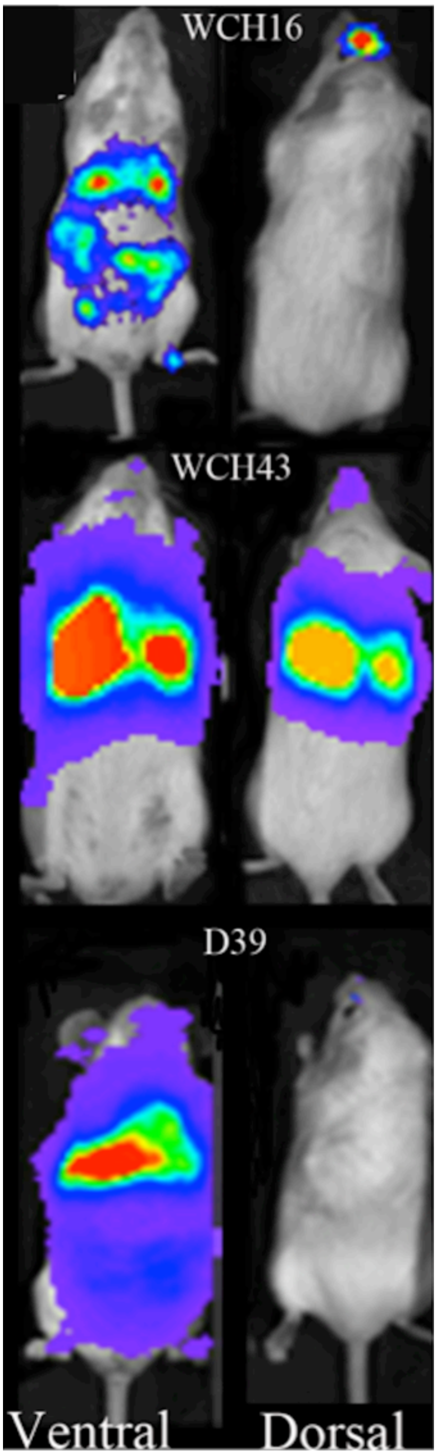

Figure S1. Bioluminescent imaging of mice infected with WCH16, WCH43 or D39 at 72 h post-challenge, showing bacteria in the nasopharynx, lungs, blood and brain, for WCH16 and WCH43, and in the nasopharynx, lungs and blood for D39.
