# Supplementary material for: A functional genomics catalogue of activated transcription factors during pathogenesis of pneumococcal disease
Source: BMC Genomics. 2014 Sep 8;15(1):769. doi: 10.1186/1471-2164-15-769 (PMC4171566; doi:10.1186/1471-2164-15-769)
Supplement: Supplementary file 2 — Additional file 2: Table S1: List of up-regulated genes of S. pneumoniae WCH16 during pathogenesis. (DOCX 163 KB) [file 12864_2014_6462_MOESM2_ESM.docx]

**Table S1.** List of up-regulated genes of *S. pneumoniae* WCH16 during pathogenesis.

| **Lungs vs Nasopharynx** | | **Blood vs Lungs** | | **Brain vs Blood** | |
| --- | --- | --- | --- | --- | --- |
| **Gene** | **Mean fold change (cut off=2.5)** | **Gene** | **Mean fold change (cut off=1.0)** | **Gene** | **Mean fold change (cut off=5.0)** |
| SP_0325 | 2.80412028 | SP_1058 | 1.427581917 | SP_0328 | 5.70209188 |
| SP_0326 | 3.117566663 | SP_1146 | 1.866524029 | SP_0333 | 6.595956618 |
| SP_0328 | 3.3475503 | SP_1431 | 1.69121229 | SP_0334 | 5.857386107 |
| SP_0336 | 2.552270082 |  |  | SP_0335 | 5.386175979 |
| SP_0342 | 2.577666069 |  |  | SP_0336 | 6.653877623 |
| SP_0393 | 3.085849871 |  |  | SP_0341 | 6.883932918 |
| SP_0421 | 4.730519702 |  |  | SP_0342 | 6.527327082 |
| SP_0422 | 3.819506787 |  |  | SP_0343 | 6.717944612 |
| SP_0424 | 4.954214213 |  |  | SP_0344 | 6.410758019 |
| SP_0429 | 3.994717816 |  |  | SP_0349 | 7.519364001 |
| SP_0430 | 3.630123479 |  |  | SP_0350 | 6.37779643 |
| SP_0431 | 2.853052494 |  |  | SP_0351 | 6.160228894 |
| SP_0432 | 4.047948229 |  |  | SP_0352 | 6.544524971 |
| SP_0435 | 2.722254161 |  |  | SP_0393 | 5.012788253 |
| SP_0437 | 4.22348466 |  |  | SP_0421 | 7.77733782 |
| SP_0438 | 3.205618893 |  |  | SP_0422 | 7.47077574 |
| SP_0439 | 3.73645364 |  |  | SP_0423 | 7.846528401 |
| SP_0440 | 4.667243479 |  |  | SP_0424 | 6.521767471 |
| SP_0445 | 3.969443627 |  |  | SP_0429 | 8.195908169 |
| SP_0446 | 3.529505799 |  |  | SP_0430 | 6.551474827 |
| SP_0447 | 3.528841024 |  |  | SP_0431 | 7.421353382 |
| SP_0448 | 3.875774251 |  |  | SP_0432 | 7.710135353 |
| SP_0589 | 2.898118026 |  |  | SP_0437 | 8.287025241 |
| SP_0655 | 4.179661469 |  |  | SP_0438 | 7.902393013 |
| SP_0656 | 3.465232663 |  |  | SP_0439 | 7.833067 |
| SP_0657 | 2.519025359 |  |  | SP_0440 | 6.690910958 |
| SP_0675 | 5.083638558 |  |  | SP_0445 | 8.013720692 |
| SP_0676 | 5.127224633 |  |  | SP_0446 | 7.579207597 |
| SP_0677 | 4.489597724 |  |  | SP_0447 | 7.454688478 |
| SP_0678 | 5.21601778 |  |  | SP_0448 | 5.933846023 |
| SP_0683 | 5.178202815 |  |  | SP_0502 | 5.223470688 |
| SP_0684 | 4.730179493 |  |  | SP_0503 | 5.011503521 |
| SP_0685 | 4.55533674 |  |  | SP_0510 | 5.164449193 |
| SP_0686 | 5.458295403 |  |  | SP_0579 | 5.097257639 |
| SP_0691 | 4.425144005 |  |  | SP_0587 | 5.075017001 |
| SP_0692 | 4.05300089 |  |  | SP_0588 | 5.570760467 |
| SP_0693 | 5.020836439 |  |  | SP_0589 | 5.134092635 |
| SP_0694 | 5.261489178 |  |  | SP_0590 | 5.236311851 |
| SP_0699 | 5.086060332 |  |  | SP_0595 | 5.491597835 |
| SP_0700 | 3.140072872 |  |  | SP_0596 | 5.865878988 |
| SP_0701 | 3.91810843 |  |  | SP_0597 | 5.363721107 |
| SP_0702 | 4.367069514 |  |  | SP_0603 | 5.485542665 |
| SP_0739 | 3.152770201 |  |  | SP_0605 | 5.003064791 |
| SP_0748 | 2.59301918 |  |  | SP_0606 | 5.015680575 |
| SP_0756 | 2.690480243 |  |  | SP_0675 | 6.588421424 |
| SP_0763 | 2.851593498 |  |  | SP_0676 | 8.073432811 |
| SP_0771 | 6.079436847 |  |  | SP_0677 | 7.934211316 |
| SP_0772 | 5.809639128 |  |  | SP_0678 | 8.010414719 |
| SP_0773 | 5.921382723 |  |  | SP_0683 | 7.535581907 |
| SP_0774 | 6.495585127 |  |  | SP_0684 | 8.153471966 |
| SP_0779 | 5.482265104 |  |  | SP_0685 | 8.015076318 |
| SP_0780 | 4.635245604 |  |  | SP_0686 | 8.103291333 |
| SP_0781 | 4.807657256 |  |  | SP_0691 | 5.486846709 |
| SP_0782 | 6.28049327 |  |  | SP_0692 | 7.982144299 |
| SP_0787 | 6.097342898 |  |  | SP_0693 | 7.299702893 |
| SP_0788 | 5.723567841 |  |  | SP_0694 | 8.12156478 |
| SP_0789 | 6.020690315 |  |  | SP_0699 | 7.410381898 |
| SP_0790 | 6.335780152 |  |  | SP_0701 | 5.242489934 |
| SP_0795 | 6.058266624 |  |  | SP_0702 | 6.08289018 |
| SP_0796 | 5.477469317 |  |  | SP_0739 | 6.514450743 |
| SP_0797 | 5.804770133 |  |  | SP_0740 | 5.764938878 |
| SP_0798 | 5.692753005 |  |  | SP_0741 | 5.614831409 |
| SP_0815 | 3.691668159 |  |  | SP_0742 | 5.89453534 |
| SP_0903 | 3.279226893 |  |  | SP_0747 | 6.622299815 |
| SP_0904 | 2.827697902 |  |  | SP_0748 | 6.213908448 |
| SP_0905 | 3.338878212 |  |  | SP_0749 | 6.068022037 |
| SP_0906 | 3.186458041 |  |  | SP_0750 | 6.769668824 |
| SP_0911 | 4.010721727 |  |  | SP_0755 | 6.995717065 |
| SP_0912 | 3.712068573 |  |  | SP_0756 | 6.239196334 |
| SP_0913 | 3.623510653 |  |  | SP_0757 | 6.57019923 |
| SP_0914 | 3.181084252 |  |  | SP_0758 | 6.549660866 |
| SP_0919 | 3.377600839 |  |  | SP_0763 | 7.099282318 |
| SP_0920 | 4.157956952 |  |  | SP_0764 | 5.907204671 |
| SP_0921 | 3.44063249 |  |  | SP_0765 | 6.537823077 |
| SP_0922 | 3.292157799 |  |  | SP_0766 | 6.663932356 |
| SP_0927 | 4.186236962 |  |  | SP_0771 | 7.368581861 |
| SP_0928 | 3.921723789 |  |  | SP_0772 | 7.343247805 |
| SP_0929 | 3.521501381 |  |  | SP_0773 | 7.03110851 |
| SP_0930 | 3.464695792 |  |  | SP_0774 | 7.351802797 |
| SP_1097 | 3.223984302 |  |  | SP_0779 | 7.586487203 |
| SP_1693 | 2.5742623 |  |  | SP_0780 | 7.319673715 |
| SP_1890 | 2.526265697 |  |  | SP_0781 | 7.177481029 |
| SP_1986 | 2.613632838 |  |  | SP_0782 | 6.790162692 |
| SP_2190 | 2.544432792 |  |  | SP_0787 | 7.528134816 |
|  |  |  |  | SP_0788 | 7.172639287 |
|  |  |  |  | SP_0789 | 7.047376513 |
|  |  |  |  | SP_0790 | 7.145609951 |
|  |  |  |  | SP_0795 | 7.430690195 |
|  |  |  |  | SP_0796 | 6.655169549 |
|  |  |  |  | SP_0797 | 6.868128444 |
|  |  |  |  | SP_0798 | 6.769231181 |
|  |  |  |  | SP_0903 | 7.293037305 |
|  |  |  |  | SP_0904 | 7.807594275 |
|  |  |  |  | SP_0905 | 7.680293143 |
|  |  |  |  | SP_0906 | 7.629169439 |
|  |  |  |  | SP_0911 | 7.835518434 |
|  |  |  |  | SP_0912 | 7.156748846 |
|  |  |  |  | SP_0913 | 7.845387097 |
|  |  |  |  | SP_0914 | 7.836796428 |
|  |  |  |  | SP_0919 | 7.631945593 |
|  |  |  |  | SP_0920 | 7.4730909 |
|  |  |  |  | SP_0921 | 7.317867067 |
|  |  |  |  | SP_0922 | 7.343912474 |
|  |  |  |  | SP_0927 | 7.657388958 |
|  |  |  |  | SP_0928 | 7.345899507 |
|  |  |  |  | SP_0929 | 7.36401289 |
|  |  |  |  | SP_0930 | 7.361879428 |
|  |  |  |  | SP_1324 | 5.202916333 |
