# Supplementary material for: A functional genomics catalogue of activated transcription factors during pathogenesis of pneumococcal disease
Source: BMC Genomics. 2014 Sep 8;15(1):769. doi: 10.1186/1471-2164-15-769 (PMC4171566; doi:10.1186/1471-2164-15-769)
Supplement: Supplementary file 3 — Additional file 3: Table S2: List of up-regulated genes of S. pneumoniae WCH43 during pathogenesis. (DOCX 168 KB) [file 12864_2014_6462_MOESM3_ESM.docx]

**Table S2.** List of up-regulated genes of *S. pneumoniae* WCH43 during pathogenesis.

| **Lungs vs Nasopharynx** | | **Blood vs Lungs** | | **Brain vs Blood** | |
| --- | --- | --- | --- | --- | --- |
| **Gene** | **Mean fold change (cut off=2.5)** | **Gene** | **Mean fold change (cut off=1.0)** | **Gene** | **Mean fold change (cut off=5.0)** |
| SP_0432 | 2.696223899 | SP_0211 | 1.541297357 | SP_0133 | 5.046687168 |
| SP_0440 | 2.792470863 | SP_0263 | 1.52798422 | SP_0225 | 6.593852628 |
| SP_0675 | 3.56559097 | SP_0538 | 1.170857394 | SP_0325 | 6.70018547 |
| SP_0676 | 3.489936912 | SP_1044 | 1.686352286 | SP_0326 | 6.735868522 |
| SP_0677 | 3.492727237 | SP_1045 | 1.095266532 | SP_0327 | 6.764307708 |
| SP_0678 | 2.88696877 | SP_1109 | 1.100955204 | SP_0328 | 7.209773032 |
| SP_0683 | 4.500410475 | SP_1329 | 2.081466055 | SP_0333 | 7.135246059 |
| SP_0684 | 3.500873133 | SP_1430 | 1.323567404 | SP_0334 | 6.580205199 |
| SP_0685 | 3.571444028 | SP_1517 | 1.414924957 | SP_0335 | 6.381831523 |
| SP_0686 | 4.06743766 | SP_1545 | 1.185059208 | SP_0336 | 6.997431391 |
| SP_0692 | 4.040717307 | SP_1673 | 1.374476767 | SP_0341 | 7.72972936 |
| SP_0693 | 3.810329294 | SP_1752 | 1.642349522 | SP_0342 | 6.726252593 |
| SP_0694 | 3.85552529 | SP_1860 | 1.551710222 | SP_0343 | 6.993855966 |
| SP_0699 | 5.297386351 | SP_2074 | 1.198766231 | SP_0344 | 7.062868265 |
| SP_0702 | 2.737607057 | SP_2182 | 1.281703253 | SP_0349 | 7.68247632 |
| SP_0771 | 6.064957227 | SP_2237 | 1.174094695 | SP_0350 | 7.288682473 |
| SP_0772 | 5.624836379 |  |  | SP_0351 | 7.107826395 |
| SP_0773 | 5.976904298 |  |  | SP_0352 | 7.593011964 |
| SP_0774 | 5.944775584 |  |  | SP_0421 | 9.878878118 |
| SP_0779 | 4.8968233 |  |  | SP_0422 | 9.104295661 |
| SP_0780 | 5.205697807 |  |  | SP_0423 | 9.199912496 |
| SP_0781 | 6.094808149 |  |  | SP_0424 | 9.105413265 |
| SP_0782 | 5.732041869 |  |  | SP_0429 | 9.786692347 |
| SP_0787 | 5.021660363 |  |  | SP_0430 | 8.986766215 |
| SP_0788 | 5.519180674 |  |  | SP_0431 | 9.106049655 |
| SP_0789 | 5.931824742 |  |  | SP_0432 | 9.766431513 |
| SP_0790 | 5.712934642 |  |  | SP_0437 | 10.48045133 |
| SP_0795 | 5.333207773 |  |  | SP_0438 | 9.452251871 |
| SP_0796 | 4.983395971 |  |  | SP_0439 | 9.382178858 |
| SP_0797 | 5.764189229 |  |  | SP_0440 | 9.723174518 |
| SP_0798 | 5.486492531 |  |  | SP_0445 | 9.999960709 |
|  |  |  |  | SP_0446 | 9.535363748 |
|  |  |  |  | SP_0447 | 9.834390302 |
|  |  |  |  | SP_0448 | 8.065379278 |
|  |  |  |  | SP_0579 | 5.465416138 |
|  |  |  |  | SP_0580 | 5.375714777 |
|  |  |  |  | SP_0581 | 5.196738905 |
|  |  |  |  | SP_0582 | 5.142399939 |
|  |  |  |  | SP_0587 | 5.653092054 |
|  |  |  |  | SP_0589 | 5.587580667 |
|  |  |  |  | SP_0590 | 5.511246614 |
|  |  |  |  | SP_0595 | 5.137151552 |
|  |  |  |  | SP_0596 | 5.246212221 |
|  |  |  |  | SP_0597 | 5.169134755 |
|  |  |  |  | SP_0603 | 5.607526934 |
|  |  |  |  | SP_0604 | 5.293607743 |
|  |  |  |  | SP_0605 | 5.370132782 |
|  |  |  |  | SP_0606 | 5.843975211 |
|  |  |  |  | SP_0675 | 9.832948247 |
|  |  |  |  | SP_0676 | 10.33216015 |
|  |  |  |  | SP_0677 | 10.31849979 |
|  |  |  |  | SP_0678 | 10.3246459 |
|  |  |  |  | SP_0683 | 9.480293783 |
|  |  |  |  | SP_0684 | 10.3852832 |
|  |  |  |  | SP_0685 | 10.18223136 |
|  |  |  |  | SP_0686 | 10.39918135 |
|  |  |  |  | SP_0691 | 8.35071926 |
|  |  |  |  | SP_0692 | 9.936195496 |
|  |  |  |  | SP_0693 | 9.988427515 |
|  |  |  |  | SP_0694 | 10.71853064 |
|  |  |  |  | SP_0699 | 9.273230035 |
|  |  |  |  | SP_0700 | 7.201789324 |
|  |  |  |  | SP_0701 | 8.588698669 |
|  |  |  |  | SP_0702 | 8.747312304 |
|  |  |  |  | SP_0739 | 7.42224968 |
|  |  |  |  | SP_0740 | 6.524913905 |
|  |  |  |  | SP_0741 | 6.85286832 |
|  |  |  |  | SP_0742 | 6.212240842 |
|  |  |  |  | SP_0747 | 6.663651726 |
|  |  |  |  | SP_0748 | 6.17299814 |
|  |  |  |  | SP_0749 | 6.241898432 |
|  |  |  |  | SP_0750 | 6.86503159 |
|  |  |  |  | SP_0755 | 6.785295859 |
|  |  |  |  | SP_0756 | 6.478179428 |
|  |  |  |  | SP_0757 | 6.148547392 |
|  |  |  |  | SP_0758 | 6.040330976 |
|  |  |  |  | SP_0763 | 7.152927932 |
|  |  |  |  | SP_0764 | 6.679542306 |
|  |  |  |  | SP_0765 | 6.523922681 |
|  |  |  |  | SP_0766 | 6.544889419 |
|  |  |  |  | SP_0771 | 9.15539918 |
|  |  |  |  | SP_0772 | 9.223139697 |
|  |  |  |  | SP_0773 | 9.361076211 |
|  |  |  |  | SP_0774 | 9.405384263 |
|  |  |  |  | SP_0779 | 9.026188792 |
|  |  |  |  | SP_0780 | 9.470730382 |
|  |  |  |  | SP_0781 | 9.506103677 |
|  |  |  |  | SP_0782 | 9.008622174 |
|  |  |  |  | SP_0787 | 9.04778389 |
|  |  |  |  | SP_0788 | 9.156748407 |
|  |  |  |  | SP_0789 | 9.098099396 |
|  |  |  |  | SP_0790 | 9.458322285 |
|  |  |  |  | SP_0795 | 8.835470089 |
|  |  |  |  | SP_0796 | 9.050732947 |
|  |  |  |  | SP_0797 | 8.955729836 |
|  |  |  |  | SP_0798 | 9.088369644 |
|  |  |  |  | SP_0885 | 5.097617508 |
|  |  |  |  | SP_0903 | 9.582514304 |
|  |  |  |  | SP_0904 | 9.992808883 |
|  |  |  |  | SP_0905 | 9.735778432 |
|  |  |  |  | SP_0906 | 9.371954574 |
|  |  |  |  | SP_0911 | 9.428603066 |
|  |  |  |  | SP_0912 | 8.860408945 |
|  |  |  |  | SP_0913 | 8.871622598 |
|  |  |  |  | SP_0914 | 9.152648652 |
|  |  |  |  | SP_0919 | 9.580426692 |
|  |  |  |  | SP_0920 | 9.24525927 |
|  |  |  |  | SP_0921 | 9.196943345 |
|  |  |  |  | SP_0922 | 8.805450701 |
|  |  |  |  | SP_0927 | 9.877442922 |
|  |  |  |  | SP_0928 | 9.370791948 |
|  |  |  |  | SP_0929 | 9.535439098 |
|  |  |  |  | SP_0930 | 9.640839027 |
|  |  |  |  | SP_1159 | 5.005438754 |
|  |  |  |  | SP_1324 | 8.177979609 |
|  |  |  |  | SP_1605 | 5.293914431 |
|  |  |  |  | SP_2111 | 5.112852409 |
