# Supplementary material for: A functional genomics catalogue of activated transcription factors during pathogenesis of pneumococcal disease
Source: BMC Genomics. 2014 Sep 8;15(1):769. doi: 10.1186/1471-2164-15-769 (PMC4171566; doi:10.1186/1471-2164-15-769)
Supplement: Supplementary file 4 — Additional file 4: Table S3: List of up-regulated genes of S. pneumoniae D39 during pathogenesis. (DOCX 155 KB) [file 12864_2014_6462_MOESM4_ESM.docx]

**Table S3.** List of up-regulated genes of *S. pneumoniae* D39 during pathogenesis.

| **Lungs vs Nasopharynx** | |
| --- | --- |
| **Gene** | **Mean fold change (cut off=1.0)** |
| SP_0236 | 1.059 |
| SP_0644 | 1.352 |
| SP_0757 | 1.0796 |
| SP_0845 | 1.492 |
| SP_0846 | 1.0979 |
| SP_1008 | 1.017 |
| SP_1101 | 1.3178 |
| SP_1110 | 1.012 |
| SP_1112 | 1.057 |
| SP_1202 | 1.077 |
| SP_1243 | 1.14 |
| SP_1530 | 1.0502 |
| SP_1647 | 1.2953 |
| SP_1711 | 1.069 |
| SP_1715 | 1.222 |
| SP_1746 | 1.167 |
| SP_1838 | 1.048 |
| SP_1939 | 1.2848 |
| SP_1966 | 1.0598 |
| SP_2030 | 1.1529 |
| SP_2070 | 1.075 |

| **Blood vs Lungs** | | **Blood vs Lungs** | |
| --- | --- | --- | --- |
| **Gene** | **Mean fold change (cut off=2.0)** | **Gene** | **Mean fold change (cut off=2.0)** |
| SP_0005 | 2.53875 | SP_1017 | 2.38375 |
| SP_0017 | 2.153 | SP_1021 | 3.0035 |
| SP_0020 | 3.244 | SP_1028 | 3.0199 |
| SP_0029 | 3.180375 | SP_1030 | 2.244 |
| SP_0033 | 3.20425 | SP_1031 | 3.76195 |
| SP_0037 | 2.22925 | SP_1036 | 2.677525 |
| SP_0059 | 3.43655 | SP_1037 | 2.628 |
| SP_0067 | 3.532825 | SP_1038 | 2.59475 |
| SP_0068 | 3.8273 | SP_1039 | 3.38425 |
| SP_0069 | 3.4761 | SP_1049 | 2.295625 |
| SP_0070 | 3.057075 | SP_1054 | 3.714875 |
| SP_0071 | 3.727025 | SP_1055 | 2.804175 |
| SP_0072 | 3.73725 | SP_1056 | 3.309575 |
| SP_0073 | 2.660925 | SP_1057 | 3.2285 |
| SP_0074 | 2.888675 | SP_1058 | 3.869875 |
| SP_0076 | 3.135375 | SP_1059 | 3.41825 |
| SP_0077 | 2.0605 | SP_1060 | 2.81925 |
| SP_0078 | 3.307725 | SP_1061 | 3.5635 |
| SP_0080 | 2.496 | SP_1062 | 3.37375 |
| SP_0090 | 2.6655 | SP_1063 | 3.247225 |
| SP_0091 | 3.169375 | SP_1064 | 3.91175 |
| SP_0092 | 3.2694 | SP_1065 | 3.37975 |
| SP_0093 | 2.5425 | SP_1066 | 2.07795 |
| SP_0094 | 3.0295 | SP_1088 | 2.4553 |
| SP_0100 | 2.525 | SP_1091 | 3.3135 |
| SP_0105 | 2.859 | SP_1095 | 3.915425 |
| SP_0108 | 2.4914 | SP_1108 | 2.95955 |
| SP_0109 | 3.267725 | SP_1109 | 3.1075 |
| SP_0114 | 3.27525 | SP_1120 | 2.28 |
| SP_0116 | 3.128875 | SP_1121 | 2.51655 |
| SP_0123 | 3.39575 | SP_1124 | 2.37305 |
| SP_0124 | 3.2394 | SP_1125 | 3.40925 |
| SP_0125 | 2.605 | SP_1126 | 3.25775 |
| SP_0126 | 2.83395 | SP_1129 | 3.865825 |
| SP_0129 | 3.644125 | SP_1130 | 3.927275 |
| SP_0130 | 2.513 | SP_1131 | 2.908 |
| SP_0131 | 2.9593 | SP_1132 | 3.2429 |
| SP_0132 | 2.360025 | SP_1133 | 3.043675 |
| SP_0133 | 2.349675 | SP_1134 | 3.337625 |
| SP_0134 | 2.24555 | SP_1135 | 3.189425 |
| SP_0139 | 2.2455 | SP_1136 | 3.0605 |
| SP_0143 | 3.562075 | SP_1137 | 3.45385 |
| SP_0147 | 2.997 | SP_1138 | 3.08875 |
| SP_0152 | 2.257125 | SP_1139 | 3.34575 |
| SP_0153 | 2.05925 | SP_1140 | 2.687325 |
| SP_0160 | 2.552175 | SP_1141 | 3.08745 |
| SP_0161 | 3.35875 | SP_1142 | 3.085225 |
| SP_0162 | 3.202 | SP_1143 | 2.914125 |
| SP_0163 | 3.889 | SP_1144 | 2.36015 |
| SP_0164 | 3.51075 | SP_1145 | 3.631 |
| SP_0165 | 3.67275 | SP_1146 | 2.9815 |
| SP_0166 | 3.365675 | SP_1147 | 2.6885 |
| SP_0167 | 2.91475 | SP_1150 | 3.5469 |
| SP_0168 | 3.93675 | SP_1158 | 3.51695 |
| SP_0169 | 3.243875 | SP_1159 | 2.0955 |
| SP_0170 | 3.407 | SP_1165 | 3.59625 |
| SP_0171 | 2.799 | SP_1170 | 2.8488 |
| SP_0172 | 2.87125 | SP_1172 | 2.7594 |
| SP_0174 | 3.2205 | SP_1173 | 2.95005 |
| SP_0179 | 2.68875 | SP_1181 | 2.559 |
| SP_0180 | 2.50725 | SP_1183 | 3.15175 |
| SP_0181 | 2.5095 | SP_1184 | 2.7386 |
| SP_0182 | 2.31125 | SP_1185 | 3.1332 |
| SP_0183 | 2.703 | SP_1186 | 2.49135 |
| SP_0188 | 3.45745 | SP_1187 | 2.92765 |
| SP_0190 | 2.569025 | SP_1188 | 3.123525 |
| SP_0194 | 2.21825 | SP_1189 | 3.5045 |
| SP_0197 | 2.3645 | SP_1194 | 3.1475 |
| SP_0200 | 2.29685 | SP_1196 | 2.5785 |
| SP_0201 | 2.984925 | SP_1197 | 2.56775 |
| SP_0241 | 3.4972 | SP_1198 | 2.7455 |
| SP_0242 | 2.7657 | SP_1199 | 2.6745 |
| SP_0243 | 3.073 | SP_1216 | 2.20255 |
| SP_0244 | 3.43525 | SP_1234 | 2.852675 |
| SP_0245 | 2.27975 | SP_1250 | 2.8 |
| SP_0249 | 2.72575 | SP_1252 | 2.54125 |
| SP_0251 | 2.0845 | SP_1253 | 2.742325 |
| SP_0257 | 3.61365 | SP_1254 | 2.466425 |
| SP_0258 | 2.616325 | SP_1256 | 2.2455 |
| SP_0265 | 2.228575 | SP_1265 | 3.27885 |
| SP_0267 | 2.0575 | SP_1292 | 3.34375 |
| SP_0269 | 2.964425 | SP_1300 | 2.325425 |
| SP_0289 | 2.68825 | SP_1302 | 2.2765 |
| SP_0296 | 3.7843 | SP_1304 | 3.063 |
| SP_0297 | 3.31295 | SP_1305 | 2.6056 |
| SP_0298 | 3.790025 | SP_1314 | 2.916875 |
| SP_0301 | 3.243 | SP_1315 | 3.61465 |
| SP_0302 | 2.74805 | SP_1316 | 3.53725 |
| SP_0303 | 3.1658 | SP_1317 | 3.0044 |
| SP_0304 | 2.699 | SP_1318 | 2.97725 |
| SP_0305 | 2.9002 | SP_1319 | 3.767875 |
| SP_0306 | 3.2855 | SP_1320 | 2.74475 |
| SP_0307 | 3.6697 | SP_1321 | 2.70585 |
| SP_0308 | 2.518 | SP_1322 | 3.7005 |
| SP_0309 | 2.81875 | SP_1323 | 2.4675 |
| SP_0310 | 2.930775 | SP_1324 | 3.2713 |
| SP_0313 | 2.1625 | SP_1325 | 3.101375 |
| SP_0315 | 3.495275 | SP_1326 | 3.7926 |
| SP_0316 | 2.84775 | SP_1327 | 3.1635 |
| SP_0317 | 3.2781 | SP_1328 | 3.396475 |
| SP_0318 | 2.8167 | SP_1329 | 2.661075 |
| SP_0319 | 2.769 | SP_1330 | 2.68525 |
| SP_0320 | 2.93925 | SP_1331 | 3.3325 |
| SP_0321 | 3.72725 | SP_1332 | 2.196675 |
| SP_0322 | 3.348075 | SP_1333 | 2.97125 |
| SP_0323 | 3.524575 | SP_1334 | 2.6455 |
| SP_0324 | 3.1805 | SP_1335 | 2.827425 |
| SP_0325 | 2.924275 | SP_1336 | 2.33305 |
| SP_0326 | 3.886425 | SP_1337 | 3.63905 |
| SP_0327 | 3.725 | SP_1338 | 2.81975 |
| SP_0328 | 3.713 | SP_1346 | 3.02 |
| SP_0329 | 2.9945 | SP_1347 | 2.8432 |
| SP_0331 | 2.6507 | SP_1348 | 3.51775 |
| SP_0332 | 3.5058 | SP_1349 | 2.76675 |
| SP_0334 | 2.2805 | SP_1350 | 3.0789 |
| SP_0338 | 2.0632 | SP_1351 | 3.0711 |
| SP_0339 | 2.585975 | SP_1352 | 3.4791 |
| SP_0343 | 3.484175 | SP_1353 | 3.2165 |
| SP_0348 | 3.44025 | SP_1379 | 2.2365 |
| SP_0349 | 2.66025 | SP_1381 | 2.02225 |
| SP_0350 | 3.033825 | SP_1418 | 3.53975 |
| SP_0351 | 3.5685 | SP_1422 | 2.4065 |
| SP_0352 | 3.599125 | SP_1423 | 2.3556 |
| SP_0353 | 3.4775 | SP_1425 | 2.0246 |
| SP_0354 | 3.47145 | SP_1428 | 2.16375 |
| SP_0355 | 3.7575 | SP_1432 | 2.11575 |
| SP_0356 | 4.062875 | SP_1439 | 3.521175 |
| SP_0357 | 3.71415 | SP_1443 | 2.59895 |
| SP_0358 | 3.370875 | SP_1446 | 2.79645 |
| SP_0359 | 3.7715 | SP_1447 | 2.181 |
| SP_0360 | 3.863575 | SP_1455 | 2.01275 |
| SP_0363 | 2.033 | SP_1487 | 2.51925 |
| SP_0365 | 2.47025 | SP_1490 | 2.56675 |
| SP_0367 | 3.01785 | SP_1492 | 2.987775 |
| SP_0379 | 3.57875 | SP_1493 | 2.1398875 |
| SP_0380 | 3.3432 | SP_1494 | 2.79625 |
| SP_0385 | 3.42975 | SP_1495 | 2.85645 |
| SP_0392 | 3.42425 | SP_1496 | 2.64325 |
| SP_0393 | 2.159625 | SP_1497 | 3.14655 |
| SP_0394 | 2.590625 | SP_1503 | 3.556625 |
| SP_0395 | 3.5036 | SP_1528 | 2.487 |
| SP_0396 | 3.68695 | SP_1562 | 2.1465 |
| SP_0397 | 3.30675 | SP_1579 | 3.02925 |
| SP_0398 | 3.23955 | SP_1581 | 2.739725 |
| SP_0399 | 3.477 | SP_1585 | 3.22625 |
| SP_0407 | 2.979775 | SP_1592 | 2.84125 |
| SP_0408 | 2.2295 | SP_1595 | 3.504575 |
| SP_0414 | 3.470425 | SP_1608 | 2.40325 |
| SP_0428 | 3.048175 | SP_1611 | 3.413875 |
| SP_0431 | 2.7687 | SP_1612 | 3.23755 |
| SP_0444 | 3.41525 | SP_1614 | 2.073325 |
| SP_0450 | 2.09675 | SP_1615 | 3.29545 |
| SP_0452 | 3.79275 | SP_1616 | 2.97535 |
| SP_0458 | 2.11825 | SP_1617 | 3.1585 |
| SP_0460 | 2.802675 | SP_1618 | 2.6357 |
| SP_0461 | 3.1419 | SP_1619 | 3.42805 |
| SP_0462 | 3.55525 | SP_1620 | 2.6208 |
| SP_0463 | 3.38045 | SP_1621 | 2.69425 |
| SP_0464 | 3.31675 | SP_1622 | 2.50025 |
| SP_0465 | 3.18925 | SP_1627 | 2.49425 |
| SP_0466 | 3.7645 | SP_1629 | 2.63875 |
| SP_0467 | 3.76225 | SP_1635 | 2.55 |
| SP_0468 | 3.576 | SP_1642 | 2.72425 |
| SP_0470 | 2.716575 | SP_1643 | 2.97825 |
| SP_0472 | 2.037825 | SP_1651 | 2.342 |
| SP_0474 | 2.509375 | SP_1657 | 2.4485 |
| SP_0475 | 2.79375 | SP_1658 | 2.44955 |
| SP_0476 | 2.77 | SP_1675 | 2.81475 |
| SP_0477 | 2.432275 | SP_1676 | 2.47625 |
| SP_0478 | 2.0335 | SP_1677 | 2.372975 |
| SP_0481 | 2.189925 | SP_1678 | 2.34175 |
| SP_0484 | 2.74575 | SP_1679 | 2.211575 |
| SP_0486 | 3.342475 | SP_1681 | 3.559775 |
| SP_0489 | 2.044275 | SP_1686 | 3.15075 |
| SP_0491 | 2.48075 | SP_1688 | 2.86945 |
| SP_0495 | 3.5944 | SP_1689 | 2.64655 |
| SP_0497 | 2.64115 | SP_1690 | 3.12175 |
| SP_0504 | 2.02265 | SP_1691 | 2.2985 |
| SP_0511 | 2.3251 | SP_1693 | 2.27575 |
| SP_0512 | 3.399875 | SP_1694 | 2.44175 |
| SP_0513 | 3.003 | SP_1703 | 3.767625 |
| SP_0514 | 2.48675 | SP_1718 | 2.986 |
| SP_0518 | 2.06125 | SP_1719 | 2.3196 |
| SP_0524 | 2.3193325 | SP_1723 | 2.15775 |
| SP_0527 | 2.02805 | SP_1736 | 2.922375 |
| SP_0528 | 2.4304575 | SP_1740 | 3.72025 |
| SP_0531 | 2.6495 | SP_1741 | 2.25575 |
| SP_0532 | 3.10375 | SP_1755 | 3.0191 |
| SP_0534 | 2.21625 | SP_1756 | 2.542225 |
| SP_0535 | 3.143875 | SP_1757 | 3.4115 |
| SP_0536 | 3.3895 | SP_1758 | 2.935825 |
| SP_0539 | 2.50965 | SP_1759 | 2.84225 |
| SP_0540 | 3.01 | SP_1760 | 2.53 |
| SP_0541 | 2.808325 | SP_1761 | 2.79175 |
| SP_0542 | 2.86725 | SP_1762 | 2.0545 |
| SP_0543 | 3.00175 | SP_1763 | 2.572225 |
| SP_0544 | 3.1908 | SP_1764 | 3.3805 |
| SP_0548 | 2.62575 | SP_1765 | 3.33595 |
| SP_0557 | 2.106425 | SP_1766 | 3.3748 |
| SP_0559 | 3.527425 | SP_1767 | 3.567475 |
| SP_0560 | 2.877875 | SP_1768 | 3.04125 |
| SP_0566 | 2.44075 | SP_1769 | 3.179325 |
| SP_0567 | 3.4622 | SP_1770 | 3.16925 |
| SP_0569 | 2.86995 | SP_1771 | 3.3282 |
| SP_0570 | 3.388875 | SP_1772 | 2.4671 |
| SP_0573 | 2.8702 | SP_1773 | 2.709575 |
| SP_0576 | 2.37725 | SP_1777 | 2.75075 |
| SP_0577 | 2.1761 | SP_1789 | 2.283775 |
| SP_0579 | 3.57975 | SP_1791 | 3.142 |
| SP_0583 | 3.0025 | SP_1794 | 2.77195 |
| SP_0584 | 3.41175 | SP_1795 | 3.143 |
| SP_0585 | 2.48475 | SP_1796 | 4.031225 |
| SP_0586 | 2.766 | SP_1797 | 3.459675 |
| SP_0594 | 2.60795 | SP_1798 | 3.15025 |
| SP_0597 | 2.182875 | SP_1799 | 3.09575 |
| SP_0598 | 2.82215 | SP_1800 | 3.16225 |
| SP_0600 | 3.427 | SP_1806 | 2.909925 |
| SP_0601 | 2.57715 | SP_1807 | 2.426075 |
| SP_0602 | 2.90975 | SP_1808 | 3.1445 |
| SP_0610 | 2.396 | SP_1811 | 2.82245 |
| SP_0619 | 2.140175 | SP_1816 | 2.03875 |
| SP_0621 | 2.058 | SP_1817 | 2.431275 |
| SP_0625 | 2.003175 | SP_1819 | 3.110625 |
| SP_0632 | 2.5993 | SP_1820 | 3.0208 |
| SP_0633 | 3.171 | SP_1822 | 3.251625 |
| SP_0643 | 2.68475 | SP_1823 | 3.183075 |
| SP_0645 | 2.44475 | SP_1824 | 2.81805 |
| SP_0646 | 2.21625 | SP_1825 | 2.45395 |
| SP_0647 | 3.69535 | SP_1827 | 3.092 |
| SP_0649 | 2.911975 | SP_1828 | 4.18 |
| SP_0650 | 2.5315 | SP_1829 | 2.1885 |
| SP_0666 | 3.1995 | SP_1830 | 3.288 |
| SP_0676 | 2.045125 | SP_1831 | 2.90575 |
| SP_0679 | 2.72215 | SP_1832 | 3.7118 |
| SP_0680 | 2.73505 | SP_1833 | 2.724 |
| SP_0683 | 3.4287 | SP_1834 | 3.645825 |
| SP_0684 | 3.226225 | SP_1835 | 3.67675 |
| SP_0685 | 2.74975 | SP_1836 | 3.335575 |
| SP_0687 | 2.26475 | SP_1842 | 2.635 |
| SP_0692 | 2.2695 | SP_1843 | 2.6615 |
| SP_0693 | 2.758775 | SP_1846 | 2.3929 |
| SP_0694 | 2.01075 | SP_1852 | 2.228 |
| SP_0696 | 2.71645 | SP_1858 | 2.0765 |
| SP_0698 | 2.17175 | SP_1864 | 2.481 |
| SP_0699 | 2.928025 | SP_1866 | 2.5055 |
| SP_0700 | 2.467 | SP_1867 | 2.75475 |
| SP_0701 | 2.399575 | SP_1868 | 3.3625 |
| SP_0702 | 2.092075 | SP_1883 | 2.42975 |
| SP_0708 | 2.23375 | SP_1884 | 3.272 |
| SP_0710 | 2.8072 | SP_1885 | 2.32425 |
| SP_0711 | 3.42125 | SP_1892 | 3.75575 |
| SP_0714 | 4.22185 | SP_1894 | 2.453 |
| SP_0716 | 2.61975 | SP_1895 | 3.1819 |
| SP_0718 | 2.64275 | SP_1896 | 3.2125 |
| SP_0719 | 2.73975 | SP_1897 | 3.2350975 |
| SP_0721 | 2.8675 | SP_1902 | 2.39775 |
| SP_0723 | 2.16775 | SP_1904 | 3.7282 |
| SP_0724 | 3.195 | SP_1908 | 3.20515 |
| SP_0725 | 2.9157 | SP_1916 | 2.523 |
| SP_0731 | 2.38485 | SP_1917 | 2.751425 |
| SP_0733 | 3.1912 | SP_1918 | 3.3481 |
| SP_0735 | 3.792875 | SP_1919 | 2.67025 |
| SP_0737 | 3.45725 | SP_1920 | 2.54185 |
| SP_0738 | 3.41775 | SP_1921 | 3.4615 |
| SP_0739 | 2.896975 | SP_1929 | 2.64875 |
| SP_0743 | 2.39475 | SP_1930 | 2.853 |
| SP_0744 | 2.8145 | SP_1934 | 3.03575 |
| SP_0747 | 3.06625 | SP_1936 | 2.62275 |
| SP_0754 | 2.61175 | SP_1962 | 2.5165 |
| SP_0759 | 2.25475 | SP_1971 | 2.4715 |
| SP_0763 | 2.394 | SP_1977 | 2.065 |
| SP_0766 | 2.326225 | SP_1995 | 2.52385 |
| SP_0772 | 3.0465 | SP_1996 | 2.0055 |
| SP_0773 | 2.5029 | SP_2003 | 2.11925 |
| SP_0774 | 3.50995 | SP_2018 | 2.79325 |
| SP_0776 | 2.0805 | SP_2025 | 3.16145 |
| SP_0783 | 3.619 | SP_2031 | 3.455425 |
| SP_0792 | 2.112675 | SP_2032 | 3.315325 |
| SP_0810 | 2.522975 | SP_2033 | 3.418 |
| SP_0811 | 3.4655 | SP_2034 | 3.3416 |
| SP_0815 | 3.068675 | SP_2035 | 3.30125 |
| SP_0817 | 2.891825 | SP_2036 | 3.456525 |
| SP_0824 | 2.048275 | SP_2037 | 3.07875 |
| SP_0826 | 3.168 | SP_2038 | 3.1835 |
| SP_0835 | 2.275225 | SP_2043 | 2.797325 |
| SP_0840 | 2.587 | SP_2046 | 2.46085 |
| SP_0854 | 2.503 | SP_2049 | 2.6783 |
| SP_0871 | 2.6555 | SP_2084 | 2.271 |
| SP_0874 | 3.879225 | SP_2085 | 2.8318 |
| SP_0875 | 3.5935 | SP_2086 | 2.446 |
| SP_0876 | 2.257 | SP_2087 | 2.82625 |
| SP_0878 | 2.27325 | SP_2088 | 2.67025 |
| SP_0879 | 2.22275 | SP_2089 | 3.1283 |
| SP_0882 | 3.1008 | SP_2090 | 3.003475 |
| SP_0883 | 2.43975 | SP_2093 | 3.29275 |
| SP_0884 | 2.8062 | SP_2104 | 2.6005 |
| SP_0885 | 2.061975 | SP_2110 | 2.0675 |
| SP_0886 | 2.042025 | SP_2116 | 2.2187 |
| SP_0888 | 2.8044 | SP_2118 | 2.0885 |
| SP_0889 | 2.67125 | SP_2119 | 2.782725 |
| SP_0890 | 4.323 | SP_2120 | 2.67075 |
| SP_0891 | 3.5824 | SP_2122 | 2.989925 |
| SP_0893 | 2.60775 | SP_2123 | 2.547325 |
| SP_0894 | 2.07725 | SP_2124 | 2.52825 |
| SP_0898 | 2.87125 | SP_2125 | 2.2803 |
| SP_0899 | 2.6635 | SP_2139 | 3.286975 |
| SP_0901 | 2.1485 | SP_2140 | 2.9702 |
| SP_0903 | 3.639925 | SP_2141 | 2.7811 |
| SP_0904 | 2.50275 | SP_2142 | 3.403 |
| SP_0906 | 2.96675 | SP_2143 | 2.496075 |
| SP_0907 | 2.66025 | SP_2144 | 3.57865 |
| SP_0908 | 2.2915 | SP_2147 | 2.097275 |
| SP_0911 | 3.13375 | SP_2154 | 2.897 |
| SP_0913 | 2.26725 | SP_2158 | 2.3781 |
| SP_0922 | 2.029025 | SP_2159 | 2.87625 |
| SP_0925 | 2.31675 | SP_2160 | 2.80515 |
| SP_0926 | 3.09925 | SP_2161 | 2.655 |
| SP_0932 | 3.80625 | SP_2163 | 2.11025 |
| SP_0934 | 3.21975 | SP_2164 | 2.99675 |
| SP_0935 | 2.1775 | SP_2165 | 2.723825 |
| SP_0940 | 3.068275 | SP_2166 | 2.39175 |
| SP_0946 | 2.88975 | SP_2178 | 2.2625 |
| SP_0949 | 2.942375 | SP_2179 | 3.48175 |
| SP_0953 | 2.064 | SP_2181 | 2.9775 |
| SP_0954 | 3.61375 | SP_2183 | 2.67575 |
| SP_0956 | 2.15775 | SP_2184 | 3.619225 |
| SP_0968 | 2.477625 | SP_2185 | 3.4815 |
| SP_0973 | 2.6785 | SP_2186 | 3.551925 |
| SP_0978 | 2.752 | SP_2190 | 3.302325 |
| SP_0997 | 2.848875 | SP_2196 | 2.0875 |
| SP_0998 | 3.00825 | SP_2199 | 2.64475 |
| SP_1005 | 2.48295 | SP_2200 | 2.9472 |
| SP_1006 | 2.9547 | SP_2207 | 2.8295 |
| SP_1007 | 2.59155 | SP_2208 | 2.922 |
| SP_1011 | 2.12225 | SP_2217 | 3.053 |
| SP_1012 | 2.30475 | SP_2231 | 2.0865 |
| SP_1016 | 2.003 | SP_2234 | 2.49625 |
