# Supplementary material for: A functional genomics catalogue of activated transcription factors during pathogenesis of pneumococcal disease
Source: BMC Genomics. 2014 Sep 8;15(1):769. doi: 10.1186/1471-2164-15-769 (PMC4171566; doi:10.1186/1471-2164-15-769)
Supplement: Supplementary file 5 — Additional file 5: Table S4: Transcription factor (TF) activation catalogue of 3 pneumococcal strains with different pathogenic profiles during infection. (DOCX 125 KB) [file 12864_2014_6462_MOESM5_ESM.docx]

**Table S4.** Transcription factor (TF) activation catalogue of 3 pneumococcal strains with different pathogenic profiles during infection.

| ***E. coli***  **TF** | ***S. pneumoniae* ortholog** | **Lungs vs Nasopharynx** | | | **Blood vs Lungs** | | | **Brain vs Blood** | | |
| --- | --- | --- | --- | --- | --- | --- | --- | --- | --- | --- |
|  |  | **WCH16** | **WCH43** | **D39** | **WCH16** | **WCH43** | **D39** | **WCH16** | **WCH43** | **D39** |
| *dnaA* | SP_0001 (*dnaA*) |  |  |  |  |  | 2 |  |  | ND*^b^* |
| *fhlA* | SP_0006 (*mfd*) | 3 |  |  |  |  | 6 | 4 | 4 | ND |
| *crp* | SP_0306 (*bglG*) | 6 | 3 | 4 |  | 2 | 70 | 8 | 11 | ND |
| *hipB* | SP_0333 (*yorfE*) | 2 | 2 |  |  | 1 | 22 | 2 | 2 | ND |
| *malT, narL, narP* | SP_0387 (*rr03*) | 1 |  | 1 |  |  | 13 | 1 | 1 | ND |
| *marR* | SP_0416 (*marR*) |  |  |  |  |  | 2 |  |  | ND |
| *nagC* | SP_0473 (*xylR*) | 4 | 1 | 1 |  | 2 | 50 | 8 | 9 | ND |
| *araC* | SP_0661 (*rr09)* |  |  | 1 |  |  | 5 |  |  | ND |
| *modE* | SP_0789 (*padR*) | 3 | 3 |  |  | 1 | 3 | 3 | 4 | ND |
| *glpR* | SP_0875 (*fruR*) |  |  | 2 |  |  | 19 | 3 | 3 | ND |
| *cynR, oxyR, cysB, gcvA, ilvY, metR* | SP_0927 (*smrC*) | 13 | 4 |  |  | 3 | 115 | 18 | 18 | ND |
| *rpoD[15, 16, 17, 18, 19], rpoE, rpoH, rpoH3, rpoS* | SP_1073 (*rpoD*) | 74 | 23 | 17 | 3 | 26 | 767 | 94 | 113 | ND |
| *ihfA* | SP_1113 (*hup*) | 26 | 10 | 4 | 2 | 4 | 249 | 36 | 39 | ND |
| *deoR* | SP_1182 (*lacR*) | 1 | 1 |  |  | 1 | 10 | 1 | 1 | ND |
| *arcA, cpxR, phoB, torR* | SP_1227 (*rr02*) | 20 | 4 | 6 | 1 | 6 | 137 | 31 | 31 | ND |
| *soxS* | SP_1433 (*araC*) | 9 | 6 |  |  |  | 26 | 11 | 11 | ND |
| *pdhR* | SP_1446 (*gntR*) | 1 | 1 |  |  |  | 1 | 1 | 1 | ND |
| *ada, fur* | SP_1463 (*ogt*) | 6 |  |  | 1 |  | 47 | 8 | 11 | ND |
| *lrp* | SP_1584 (*codY*) | 19 | 3 |  |  | 8 | 175 | 28 | 25 | ND |
| *tyrR* | SP_1654 (HP)*^a^* | 5 |  | 2 |  | 1 | 71 | 11 | 13 | ND |
| *metJ* | SP_1694 (HP)*^a^* | 3 |  | 1 |  |  | 19 | 3 | 4 | ND |
| *fnr* | SP_1697 (*recG*) | 9 | 6 | 2 |  | 4 | 61 | 11 | 17 | ND |
| *purR* | SP_1725 (*scrR*) | 16 | 4 | 1 |  | 1 | 72 | 15 | 17 | ND |
| *lacI* | SP_1799 (*lacI*) | 3 |  |  |  | 1 |  | 3 | 3 | ND |
| *galR* | SP_1854 (*galR*) |  |  |  |  |  | 1 |  |  | ND |
| *farR* | SP_1885 (*treR*) |  |  | 1 |  | 1 | 1 |  |  | ND |
| *melR* | SP_1899 (*msmR*) |  |  |  |  |  | 1 |  |  | ND |
| *rpoN* | SP_1963 (HP)*^a^* |  |  |  |  |  | 2 |  |  | ND |
| *cytR* | SP_1999 (*ccpA*) | 2 | 1 | 1 |  |  | 9 | 1 | 1 | ND |
| *argR* | SP_2077 (*argR*) | 14 | 7 | 1 | 4 | 7 | 201 | 17 | 23 | ND |
| *ompR* | SP_2082 (*pnpR*) | 5 | 5 | 1 |  |  | 39 | 7 | 7 | ND |
|  | **TOTAL** | **245** | **84** | **46** | **11** | **69** | **2196** | **325** | **369** | ND |

*^a^* HP=Hypothetical protein

*^b^* ND= Not determined.
