# Supplementary material for: A functional genomics catalogue of activated transcription factors during pathogenesis of pneumococcal disease
Source: BMC Genomics. 2014 Sep 8;15(1):769. doi: 10.1186/1471-2164-15-769 (PMC4171566; doi:10.1186/1471-2164-15-769)
Supplement: Supplementary file 6 — Additional file 6: Table S5.: List of highly activated transcription factors (TFs) during pathogenesis of S. pneumoniae WCH16, WCH43, and D39 and their potential regulatory elements. (DOCX 86 KB) [file 12864_2014_6462_MOESM6_ESM.docx]

**Table S5.** List of highly activated transcription factors (TFs) during pathogenesis of *S. pneumoniae* WCH16, WCH43, and D39 and their potential regulatory elements.

| **TF** | **Regulatory elements (TFBs)*^*^*** |
| --- | --- |
| SP_0927 (*smrC*) | ATATATCA/TTATATTT/TATAGTTA/TTTTTTCA/TTTATTAA/ATTAGTGT/TAGAATAG/TAAGGTAA |
| SP_1073 (*rpoD*) | TTTTTTTT/AAATAATG/TTTTGTTT/TAAGGTTA/GAGTATAA/TTATAAAA/TTTTTATA/TGATATAA/TAATTAGA/CGTATAAT/AGTGTAAT/TGTTATAA/ATAATAAT/TTTTACTT/ACAATCTT/TTTTATAG/TTTATAAT/TGATAATT/TTTTGTAT/ATACTATA/TGTAAAAT/AATAAATA/TTTGTTTT/TTGTCTGA/TATAGATT |
| SP_1113 (*hup*) | AAATAAAA/TTTTTAAG/AATAAAAT/TGTAAGAA/TTTTATTT/ACAAAAAA/TTAATTTA/ACAAAAAA/TGATATGA/AGATATAT/TATACTTT/TTTCAAAA/TTTTATTT |
| SP_1227 (*rr02*) | AATAAAAG/ATGTAACA/TTTTCATA/TCATAAAA/CTTGTAAG/TAAAAAGA/TAAAATTA/AATAAAAA/TAACAATT/TAATTAAA/AATAAAAA/ATTTGTAA/AATAAAAA |
| SP_1584 (*codY*) | TATTTATT/ATTTATTA/ATCTTTTA/TTTCTTTT/TATTTTTT/ATTTTTTT/TGCATTTT |
| SP_1725 (*scrR*) | ATAAAAAG/TAATCGTT/TTTTTAAG |
| SP_2077 (*argR*) | TTTTTTAT/TTTTTATT/ACATATAA/CATATTTT/ATATAAAT/CATAAAAA/TTTATTTT/TTATAATT/ATAAAAAT |

* Orthologous TFBs were extracted from *E. coli*.
