# Supplementary material for: A functional genomics catalogue of activated transcription factors during pathogenesis of pneumococcal disease
Source: BMC Genomics. 2014 Sep 8;15(1):769. doi: 10.1186/1471-2164-15-769 (PMC4171566; doi:10.1186/1471-2164-15-769)
Supplement: Supplementary file 7 — Additional file 7: Table S6: Validation of TF activation maps for S. pneumoniae WCH16, WCH43 and D39, and for all 3 strains and validation of the clustering profiles of activated TFs of all 3 S. pneumoniae strains across niches (Figures 2A, 2B, 2C, 2D and Figure 3). (DOCX 32 KB) [file 12864_2014_6462_MOESM7_ESM.docx]

**Table S6.** Validation of TF activation maps for *S. pneumoniae* WCH16, WCH43 and D39, and for all 3 strains and validation of the clustering profiles of activated TFs of all 3 *S. pneumoniae* strains across niches (Figures 2A, 2B, 2C, 2D and Figure 3).

Validation of Figure 2A (TF activation map for *S. pneumoniae* WCH16).

| **Internal validation measures** | | | | **Stability validation measures** | | | |
| --- | --- | --- | --- | --- | --- | --- | --- |
| Score | Value | Method | Cluster number | Score | Value | Method | Cluster number |
| Connectivity | 2.9290 | hierarchical | 2 | APN | 0.000 | hierarchical | 2 |
| Dunn | Infinite | hierarchical | 20 | AD | 0.000 | hierarchical | 29 |
| Silhouette | 0.8494 | hierarchical | 2 | ADM | 0.000 | hierarchical | 2 |
|  |  |  |  | FOM | 0.000 | hierarchical | 29 |

Validation of Figure 2B (TF activation map for *S. pneumoniae* WCH43).

| **Internal validation measures** | | | | **Stability validation measures** | | | |
| --- | --- | --- | --- | --- | --- | --- | --- |
| Score | Value | Method | Cluster number | Score | Value | Method | Cluster number |
| Connectivity | 2.9290 | hierarchical | 2 | APN | 0.000 | hierarchical | 2 |
| Dunn | Infinite | hierarchical | 24 | AD | 0.043 | hierarchical | 30 |
| Silhouette | 0.8579 | hierarchical | 2 | ADM | 0.000 | hierarchical | 2 |
|  |  |  |  | FOM | 0.631 | hierarchical | 28 |

Validation of Figure 2C (TF activation map for *S. pneumoniae* D39).

| **Internal validation measures** | | | | **Stability validation measures** | | | |
| --- | --- | --- | --- | --- | --- | --- | --- |
| Score | Value | Method | Cluster number | Score | Value | Method | Cluster number |
| Connectivity | 2.9290 | hierarchical | 2 | APN | 0.0000 | hierarchical | 2 |
| Dunn | Infinite | hierarchical | 27 | AD | 0.0645 | hierarchical | 30 |
| Silhouette | 0.8749 | hierarchical | 2 | ADM | 0.0000 | hierarchical | 2 |
|  |  |  |  | FOM | 1.4142 | hierarchical | 30 |

Validation of Figure 2D (TF activation map for all 3 *S. pneumoniae* strains).

| **Internal validation measures** | | | | **Stability validation measures** | | | |
| --- | --- | --- | --- | --- | --- | --- | --- |
| Score | Value | Method | Cluster number | Score | Value | Method | Cluster number |
| Connectivity | 2.9290 | hierarchical | 2 | APN | 0.000 | hierarchical | 2 |
| Dunn | Infinite | hierarchical | 28 | AD | 0.000 | hierarchical | 30 |
| Silhouette | 0.8731 | hierarchical | 2 | ADM | 0.000 | hierarchical | 2 |
|  |  |  |  | FOM | 0.000 | hierarchical | 30 |

Validation of Figure 3 (**Clustering profiles of activated transcription factors of all 3 *S. pneumoniae* strains across niches).**

|  | Number of clusters | | | | | | |
| --- | --- | --- | --- | --- | --- | --- | --- |
| **Internal validation measures** | **2** | **3** | **4** | **5** | **6** | **7** | **8** |
| Connectivity | 2.9290 | 8.6167 | 11.4500 | 13.9667 | 19.1603 | 21.1603 | 23.1603 |
| Dunn | 2.0745 | 0.3144 | 0.5009 | 0.5127 | 0.3989 | 0.3989 | 0.3989 |
| Silhouette | 0.8731 | 0.7249 | 0.6572 | 0.6070 | 0.6383 | 0.5937 | 0.5703 |
| **Stability validation measures** |  |  |  |  |  |  |  |
| Average proportion of non-overlap (APN) | 0.0000 | 0.0097 | 0.0229 | 0.0229 | 0.0272 | 0.0272 | 0.0191 |
| Average distance (AD) | 62.8252 | 33.2411 | 28.6954 | 25.5315 | 12.7774 | 11.4004 | 10.1764 |
| Average distance between means (ADM) | 0.0000 | 2.3882 | 2.0207 | 1.7740 | 1.5277 | 1.2982 | 0.7855 |
| Figure of merit (FOM) | 12.4686 | 8.3392 | 6.5388 | 6.1864 | 4.9034 | 3.4611 | 3.1881 |
|  |  |  |  |  |  |  |  |
|  | **Optimal Scores** | | | | | | |
| **Optimal Scores:**  **Internal validation measures** | Score | Method | Cluster number |  |  |  |  |
| Connectivity | 2.9290 | hierarchical | 2 |  |  |  |  |
| Dunn | 2.0745 | hierarchical | 2 |  |  |  |  |
| Silhouette | 0.8731 | hierarchical | 2 |  |  |  |  |
| **Optimal Scores:**  **Stability validation measures** | Score | Method | Cluster number |  |  |  |  |
| APN | 0.0000 | hierarchical | 2 |  |  |  |  |
| AD | 10.1764 | hierarchical | 8 |  |  |  |  |
| ADM | 0.0000 | hierarchical | 2 |  |  |  |  |
| FOM | 3.1881 | hierarchical | 8 |  |  |  |  |
