# Supplementary material for: A functional genomics catalogue of activated transcription factors during pathogenesis of pneumococcal disease
Source: BMC Genomics. 2014 Sep 8;15(1):769. doi: 10.1186/1471-2164-15-769 (PMC4171566; doi:10.1186/1471-2164-15-769)
Supplement: Supplementary file 8 — Additional file 8: Table S7: Comparative discriminant function analysis of Streptococcus pneumoniae transcription factors during pathogenesis. (DOCX 52 KB) [file 12864_2014_6462_MOESM8_ESM.docx]

**Table S7.** Comparative discriminant function analysis of *Streptococcus pneumoniae* transcription factors during pathogenesis.

***Part A. WCH16 versus WCH43***

| **Transition from Nasopharynx to Lungs** | | | | | **Transition from Lungs to Blood** | | | | | **Transition from Blood to Brain** | | | | |
| --- | --- | --- | --- | --- | --- | --- | --- | --- | --- | --- | --- | --- | --- | --- |
| Transcription factor | | WCH16 coefficient | WCH43 coefficient | Absolute coefficient difference | Transcription factor | | WCH16 coefficient | WCH43 coefficient | Absolute coefficient difference | Transcription factor | | WCH16 coefficient | WCH43 coefficient | Absolute coefficient difference |
| **SP_0927** | (***smrC***) | **1.141** | **1.842** | **0.701** | **SP_0927** | (***smrC***) | **0** | **2.833** | **2.833** | **SP_0927** | (***smrC***) | **0.7324** | **0.6359** | **0.0965** |
| **SP_1227** | (***rr02***) | **-0.467** | **-0.157** | **0.31** | **SP_1227** | (***rr02***) | **0** | **4.25** | **4.25** | **SP_1227** | (***rr02***) | **1.0698** | **0.922** | **0.1478** |
| **SP_1113** | (***hup***) | **0.641** | **0.615** | **0.026** | **SP_1113** | (***hup***) | **17** | **-7.083** | **24.083** | **SP_1113** | (***hup***) | **0.5144** | **0.3148** | **0.1996** |
| **SP_2077** | (***argR***) | **0.656** | **1.106** | **0.45** | **SP_2077** | (***argR***) | **0** | **1.417** | **1.417** | **SP_2077** | (***argR***) | **0.3931** | **0.5809** | **0.1878** |
| **SP_1584** | (***codY***) | **1.298** | **0.674** | **0.624** | **SP_1584** | (***codY***) | **-38.25** | **13.458** | **51.708** | **SP_1584** | (***codY***) | **0.76** | **0.703** | **0.057** |
| **SP_1725** | (***scrR***) | **-0.224** | **-0.837** | **0.613** | **SP_1725** | (***scrR***) | **0** | **-11.333** | **11.333** | **SP_1725** | (***scrR***) | **-0.9376** | **-0.7941** | **0.1435** |
| **SP_1073** | (***rpoD***) | **1.846** | **1.6** | **0.246** | **SP_1073** | (***rpoD***) | **0** | **1.417** | **1.417** | **SP_1073** | (***rpoD***) | **1.0346** | **1.1196** | **0.085** |
| SP_1697 | (*recG*) | 1.713 | 2.84 | 1.127 | SP_1697 | (*recG*) | 51 | -24.083 | 75.083 | SP_1697 | (*recG*) | 0.8541 | 1.2525 | 0.3984 |
| SP_2082 | (*pnpR*) | 2.629 | 4.146 | 1.517 | SP_0473 | (*xylR*) | 25.5 | -5.667 | 31.167 | SP_2082 | (*pnpR*) | 1.2585 | 1.4688 | 0.2103 |
| SP_0473 | (*xylR*) | -5.345 | -7.814 | 2.469 | SP_0306 | (*bglG*) | -17 | 7.083 | 24.083 | SP_0473 | (*xylR*) | -0.6015 | -0.975 | 0.3735 |
| SP_1433 | (*araC*) | -15.509 | -16.278 | 0.769 | SP_1799 | (*LacI*) | 17 | -5.667 | 22.667 | SP_1433 | (*araC*) | -0.7899 | -0.7561 | 0.0338 |
| SP_0333 | (*yorfE*) | 18.003 | 19.7 | 1.697 | SP_1182 | (*lacR*) | 0 | -1.417 | 1.417 | SP_0333 | (*yorfE*) | 3.4685 | 3.268 | 0.2005 |
| SP_1999 | (*ccpA*) | 2.794 | 3.276 | 0.482 | SP_1654 | (*HP*) | 0 | 0 | 0 | SP_1999 | (*ccpA*) | 3.0717 | 3.0927 | 0.021 |
| SP_0306 | (*bglG*) | -1.808 | 0.225 | 1.583 | SP_1885 | (*treR*) | 0 | 2.833 | 2.833 | SP_0306 | (*bglG*) | 0.1717 | 0.4893 | 0.3176 |
| SP_1799 | (*LacI*) | 18.795 | 18.035 | 0.76 | **Constant** |  | **-5.667** | **-1.594** |  | SP_1799 | (*LacI*) | 3.3472 | 3.5341 | 0.1869 |
| SP_1694 | (*HP*) | 3.928 | 2.372 | 1.556 | **Average of absolute mean of coefficient difference** |  |  |  | **18.16** | SP_1694 | (*HP*) | 2.4806 | 2.7868 | 0.3062 |
| SP_0789 | (*padR*) | 18.658 | 20.806 | 2.148 |  |  |  |  |  | SP_0789 | (*padR*) | 3.4583 | 3.7266 | 0.2683 |
| SP_1654 | (*HP*) | -6.244 | -7.751 | 1.507 |  |  |  |  |  | SP_1182 | (*lacR*) | -7.3217 | -9.3532 | 2.0315 |
| SP_1463 | (*ogt*) | 8.168 | 7.375 | 0.793 |  |  |  |  |  | SP_1654 | (*HP*) | 0.6812 | 0.7544 | 0.0732 |
| SP_0006 | (*mfd*) | -3.768 | -4.055 | 0.287 |  |  |  |  |  | SP_1463 | (*ogt*) | -1.9156 | -1.2713 | 0.6443 |
| SP_0387 | (*rr03*) | -1.095 | -3.618 | 2.523 |  |  |  |  |  | SP_0875 | (*fruR*) | 1.8596 | 2.0049 | 0.1453 |
| **Constant** |  | **-1.2** | **-1.5** |  |  |  |  |  |  | SP_0006 | (*mfd*) | 2.1039 | 1.4108 | 0.6931 |
| **Average of absolute mean of coefficient difference** |  |  |  | **1.01** |  |  |  |  |  | SP_0387 | (*rr03*) | -2.2273 | -2.405 | 0.1777 |
|  |  |  |  |  |  |  |  |  |  | **Constant** |  | **-1.0026** | **-1.1031** |  |
|  |  |  |  |  |  |  |  |  |  | **Average of absolute mean of coefficient difference** |  |  |  | **0.3** |

***Part B. D39 versus WCH16***

| **Transition from Nasopharynx to Lungs** | | | | | **Transition from to Lungs to Blood** | | | | |
| --- | --- | --- | --- | --- | --- | --- | --- | --- | --- |
| Transcription factor | | D39 coefficient | WCH16  coefficient | Absolute coefficient difference | Transcription factor | | D39 coefficient | WCH16 coefficient | Absolute coefficient difference |
| **SP_0927** | (***smrC***) | **-0.088** | **1.889** | **1.977** | **SP_0927** | (***smrC***) | **0.6601** | **-0.0344** | **0.6945** |
| **SP_1227** | (***rr02***) | **2.888** | **-0.059** | **2.947** | **SP_1227** | (***rr02***) | **0.2086** | **0.4003** | **0.1917** |
| **SP_1113** | (***hup***) | **0.472** | **0.764** | **0.292** | **SP_1113** | (***hup***) | **0.4399** | **1.5839** | **1.144** |
| **SP_2077** | (***argR***) | **0.147** | **0.89** | **0.743** | **SP_2077** | (***argR***) | **0.2023** | **3.063** | **2.8607** |
| **SP_1584** | (***codY***) | **0.524** | **1.205** | **0.681** | **SP_1584** | (***codY***) | **-0.0704** | **-1.676** | **1.6056** |
| **SP_1725** | (***scrR***) | **-3.406** | **0.371** | **3.777** | **SP_1725** | (***scrR***) | **0.6998** | **-1.0256** | **1.7254** |
| **SP_1073** | (***rpoD***) | **2.269** | **1.273** | **0.996** | **SP_1073** | (***rpoD***) | **0.3119** | **0.12** | **0.1919** |
| SP_1697 | (*recG*) | 3.835 | 1.727 | 2.108 | SP_1697 | (*recG*) | 0.4724 | -1.557 | 2.0294 |
| SP_2082 | (*pnpR*) | 1.335 | 2.853 | 1.518 | SP_2082 | (*pnpR*) | 0.9751 | 1.1538 | 0.1787 |
| SP_0473 | (*xylR*) | -7.365 | -5.764 | 1.601 | SP_0473 | (*xylR*) | 0.2898 | -0.535 | 0.8248 |
| SP_1433 | (*araC*) | -17.691 | -13.172 | 4.519 | SP_1433 | (*araC*) | 1.0955 | -0.2295 | 1.325 |
| SP_0333 | (*yorfE*) | 19.62 | 15.918 | 3.702 | SP_0333 | (*yorfE*) | 0.3074 | 0.2949 | 0.0125 |
| SP_1999 | (*ccpA*) | 0.941 | 2.999 | 2.058 | SP_1999 | (*ccpA*) | 0.8296 | -0.3775 | 1.2071 |
| SP_0306 | (*bglG*) | -2.043 | -0.805 | 1.238 | SP_0306 | (*bglG*) | 0.7366 | -0.0173 | 0.7539 |
| SP_1799 | (*LacI*) | 19.295 | 16.044 | 3.251 | SP_1694 | (*HP*) | 1.5509 | 0.7153 | 0.8356 |
| SP_1694 | (*HP*) | 2.076 | 3.636 | 1.56 | SP_0789 | (*padR*) | 1.1862 | -0.3021 | 1.4883 |
| SP_0789 | (*padR*) | 19.767 | 16.807 | 2.96 | SP_1182 | (*lacR*) | 0.7004 | 0.4036 | 0.2968 |
| SP_1654 | (*HP*) | -8.046 | -5.209 | 2.837 | SP_1446 | (*gntR*) | 0.5893 | -1.7911 | 2.3804 |
| SP_1463 | (*ogt*) | 2.212 | 4.726 | 2.514 | SP_1654 | (*HP*) | 0.6493 | -0.1835 | 0.8328 |
| SP_0006 | (*mfd*) | 2.555 | -0.229 | 2.784 | SP_1463 | (*ogt*) | 0.8679 | 2.8643 | 1.9964 |
| SP_0387 | (*rr03*) | -5.408 | -1.63 | 3.778 | SP_0875 | (*fruR*) | 0.2469 | -1.0104 | 1.2573 |
| **Constant** |  | **-2.221** | **-1.246** |  | SP_0006 | (*mfd*) | -0.7168 | -0.542 | 0.1748 |
| **Average of absolute mean of coefficient difference** |  |  |  | **2.278142857** | SP_0387 | (*rr03*) | 1.0194 | 1.1176 | 0.0982 |
|  |  |  |  |  | SP_1885 | (*treR*) | 1.0648 | -0.9237 | 1.9885 |
|  |  |  |  |  | SP_0661 | (*rr09*) | 0.5065 | 0.0431 | 0.4634 |
|  |  |  |  |  | SP_1963 | (*HP*) | 1.4882 | -2.6126 | 4.1008 |
|  |  |  |  |  | SP_0416 | (*marR*) | 0.0744 | 0.9656 | 0.8912 |
|  |  |  |  |  | SP_1854 | (*galR*) | 0.3623 | -1.7333 | 2.0956 |
|  |  |  |  |  | SP_0001 | (*dnaA*) | 1.5421 | 1.251 | 0.2911 |
|  |  |  |  |  | SP_1899 | (*msmR*) | 1.9668 | 1.1137 | 0.8531 |
|  |  |  |  |  | **Constant** |  | **-0.6386** | **-3.1741** |  |
|  |  |  |  |  | **Average of absolute mean of coefficient difference** |  |  |  | **1.204032** |

***Part C. D39 versus WCH43***

| **Transition from Nasopharynx to Lungs** | | | | | **Transition from Lungs to Blood** | | | | |
| --- | --- | --- | --- | --- | --- | --- | --- | --- | --- |
| Transcription factor | | D39 coefficient | WCH43  coefficient | Absolute coefficient difference | Transcription factor | | D39  coefficient | WCH43 coefficient | Absolute coefficient difference |
| **SP_0927** | (***smrC***) | **-0.86** | **3.184** | **4.044** | **SP_0927** | (***smrC***) | **0.681** | **0.726** | **0.045** |
| **SP_1227** | (***rr02***) | **1.615** | **-0.628** | **2.243** | **SP_1227** | (***rr02***) | **0.193** | **1.23** | **1.037** |
| **SP_1113** | (***hup***) | **0.636** | **1.14** | **0.504** | **SP_1113** | (***hup***) | **0.469** | **-0.222** | **0.691** |
| **SP_2077** | (***argR***) | **-0.999** | **0.651** | **1.65** | **SP_2077** | (***argR***) | **0.191** | **0.071** | **0.12** |
| **SP_1584** | (***codY***) | **1.499** | **-1.543** | **3.042** | **SP_1584** | (***codY***) | **-0.138** | **0.769** | **0.907** |
| **SP_1725** | (***scrR***) | **-1.227** | **0.703** | **1.93** | **SP_1725** | (***scrR***) | **0.72** | **-0.177** | **0.897** |
| **SP_1073** | (***rpoD***) | **2.774** | **1.237** | **1.537** | **SP_1073** | (***rpoD***) | **0.315** | **0.261** | **0.054** |
| SP_1697 | (*recG*) | 2.856 | 1.802 | 1.054 | SP_1697 | (*recG*) | 0.321 | 1.805 | 1.484 |
| SP_2082 | (*pnpR*) | 0.109 | 3.813 | 3.704 | SP_2082 | (*pnpR*) | 1.034 | -0.639 | 1.673 |
| SP_0473 | (*xylR*) | -4.546 | -4.637 | 0.091 | SP_0473 | (*xylR*) | 0.277 | 1.621 | 1.344 |
| SP_1433 | (*araC*) | -22.158 | -15.079 | 7.079 | SP_1433 | (*araC*) | 0.999 | 0.808 | 0.191 |
| SP_0333 | (*yorfE*) | 25.071 | 18.848 | 6.223 | SP_0333 | (*yorfE*) | 0.325 | -0.336 | 0.661 |
| SP_1999 | (*ccpA*) | 1.914 | 4.421 | 2.507 | SP_1999 | (*ccpA*) | 0.81 | -0.256 | 1.066 |
| SP_0306 | (*bglG*) | -3.634 | 1.946 | 5.58 | SP_0306 | (*bglG*) | 0.75 | 0.531 | 0.219 |
| SP_0789 | (*padR*) | 24.072 | 19.5 | 4.572 | SP_1799 | (*LacI*) | -0.125 | 53.053 | 53.178 |
| **Constant** |  | **-2.245** | **-1.517** |  | SP_1694 | (*HP*) | 1.556 | 0.409 | 1.147 |
| **Average of absolute mean of coefficient difference** |  |  |  | **3.05** | SP_0789 | (*padR*) | 0.007 | 10.203 | 10.196 |
|  |  |  |  |  | SP_1182 | (*lacR*) | 0.702 | 0.265 | 0.437 |
|  |  |  |  |  | SP_1446 | (*gntR*) | 0.597 | -2.735 | 3.332 |
|  |  |  |  |  | SP_1654 | (*HP*) | 0.643 | 0.641 | 0.002 |
|  |  |  |  |  | SP_1463 | (*ogt*) | 0.906 | -0.037 | 0.943 |
|  |  |  |  |  | SP_0875 | (*fruR*) | 0.244 | -0.324 | 0.568 |
|  |  |  |  |  | SP_0006 | (*mfd*) | -0.695 | -1.989 | 1.294 |
|  |  |  |  |  | SP_0387 | (*rr03*) | 1.008 | 0.517 | 0.491 |
|  |  |  |  |  | SP_1885 | (*treR*) | 0.995 | 27.472 | 26.477 |
|  |  |  |  |  | SP_0661 | (*rr09*) | 0.538 | -0.758 | 1.296 |
|  |  |  |  |  | SP_1963 | (*HP*) | 1.476 | 1.029 | 0.447 |
|  |  |  |  |  | SP_0416 | (*marR*) | 0.097 | -0.834 | 0.931 |
|  |  |  |  |  | SP_1854 | (*galR*) | 0.305 | 0.499 | 0.194 |
|  |  |  |  |  | SP_0001 | (*dnaA*) | 1.526 | 0.723 | 0.803 |
|  |  |  |  |  | SP_1899 | (*msmR*) | 1.961 | 0.993 | 0.968 |
|  |  |  |  |  | **Constant** |  | **-0.628** | **-4.153** |  |
|  |  |  |  |  | **Average of absolute mean of coefficient difference** |  |  |  | **3.648** |
