# Supplementary material for: A functional genomics catalogue of activated transcription factors during pathogenesis of pneumococcal disease
Source: BMC Genomics. 2014 Sep 8;15(1):769. doi: 10.1186/1471-2164-15-769 (PMC4171566; doi:10.1186/1471-2164-15-769)
Supplement: Supplementary file 9 — Additional file 9: Table S8: Genes co-regulated by the same Transcription factors (TFs) during pneumococcal pathogenesis [SP_0927 (smrC), SP_1073 (rpoD), SP_1113 (hup), SP_1227 (rr02), SP_1584 (codY), SP_1725 (scrR), and SP_2077 (argR)]. (DOCX 152 KB) [file 12864_2014_6462_MOESM9_ESM.docx]

**Table S8**. Genes co-regulated by the same Transcription factors (TFs) during pneumococcal pathogenesis [SP_0927 (*smrC*), SP_1073 (*rpoD*), SP_1113 (*hup*), SP_1227 (*rr02*), SP_1584 (*codY*), SP_1725 (*scrR*), and SP_2077 (*argR*)].

| **Gene** | **Lungs vs Nasopharynx** | | | **Blood vs Lungs** | | | **Brain vs Blood** | |
| --- | --- | --- | --- | --- | --- | --- | --- | --- |
|  | **WCH16** | **WCH43** | **D39** | **WCH16** | **WCH43** | **D39** | **WCH16** | **WCH43** |
| SP_0693 | *codY, hup, rpoD, smrC* | *codY, hup, rpoD, smrC* |  |  |  | *codY, hup, rpoD, smrC* | *codY, hup, rpoD, smrC* | *codY, hup, rpoD, smrC* |
| SP_0904-SP_0906 | *hup, rpoD, rr02, scrR, smrC* |  |  |  |  |  | *hup, rpoD, rr02, scrR, smrC* | *hup, rpoD, rr02, scrR, smrC* |
| SP_0914 | *argR, rpoD, rr02, smrC* |  |  |  |  |  | *argR, rpoD, rr02, smrC* | *argR, rpoD, rr02, smrC* |
| SP_0421-SP_0431 | *hup, rpoD rr02, scrR* |  |  |  |  |  | *hup, rpoD rr02, scrR* | *hup, rpoD rr02, scrR* |
| SP_0686 | *hup, rpoD, rr02, scrR* | *hup, rpoD, rr02, scrR* |  |  |  |  | *hup, rpoD, rr02, scrR* | *hup, rpoD, rr02, scrR* |
| SP_0325-SP_0326 | *argR, hup, rpoD, scrR* |  |  |  |  |  |  |  |
| SP_0325-SP_0327 |  |  |  |  |  |  |  | *argR, hup, rpoD, scrR* |
| SP_0393 | *argR, hup, rpoD, scrR* |  |  |  |  |  | *argR, hup, rpoD, scrR* |  |
| SP_0698 | *argR, rpoD, rr02, scrR* | *argR, rpoD, rr02, scrR* |  |  |  |  | *argR, rpoD, rr02, scrR* | *argR, rpoD, rr02, scrR* |
| SP_1058 |  |  |  | *argR, hup, rpoD, rr02* |  |  |  |  |
| SP_0502 |  |  |  |  |  |  | *codY, hup, rpoD, rr02* |  |
| SP_0758 |  |  |  |  |  |  | *argR, codY, hup, rpoD, rr02* | *argR, codY, hup, rpoD, rr02* |
| SP_1329 |  |  |  |  | *codY, hup, rpoD, smrC* | *codY, hup, rpoD, smrC* |  |  |
| SP_0263 |  |  |  |  | *argR, codY, rpoD, rr02* |  |  |  |
| SP_2182 |  |  |  |  | *argR, codY, rpoD, rr02* |  |  |  |
| SP_1324 |  |  |  |  |  |  |  | *argR, codY, hup, rpoD, smrC* |
| SP_1647 |  |  | *argR, hup, rpoD, rr02* |  |  |  |  |  |
| SP_0005 |  |  |  |  |  | *argR, codY, hup, scrR* |  |  |
| SP_0105 |  |  |  |  |  | *argR, codY, hup, rpoD* |  |  |
| SP_0116 |  |  |  |  |  | *codY, rpoD, rr02, smrC* |  |  |
| SP_0124-SP_0125 |  |  |  |  |  | *argR, codY, hup, rpoD* |  |  |
| SP_0379-SP_0380 |  |  |  |  |  | *hup, rpoD, rr02, smrC* |  |  |
| SP_0385 |  |  |  |  |  | *argR, hup, rpoD, scrR* |  |  |
| SP_0394-SP_0397 |  |  |  |  |  | *argR, hup, rpoD, scrR* |  |  |
| SP_0428 |  |  |  |  |  | *hup, rpoD, rr02, scrR* |  |  |
| SP_0431 |  |  |  |  |  | *hup, rpoD, rr02, scrR* |  |  |
| SP_0585-SP_0586 |  |  |  |  |  | *codY, hup, rpoD, smrC* |  |  |
| SP_0714 |  |  |  |  |  | *codY, rpoD, rr02, smrC* |  |  |
| SP_0874 |  |  |  |  |  | *argR, codY, hup, rpoD, smrC* |  |  |
| SP_0893-SP_0894 |  |  |  |  |  | *argR, rpoD, rr02, smrC* |  |  |
| SP_0904_SP_0906, SP_0907 |  |  |  |  |  | *hup, rpoD, rr02, scrR, smrC* |  |  |
| SP_1036 |  |  |  |  |  | *argR, codY, hup, rpoD, smrC* |  |  |
| SP_1315-SP_1331 |  |  |  |  |  | *codY, hup, rpoD, smrC* |  |  |
| SP_1658 |  |  |  |  |  | *argR, hup, rpoD, smrC* |  |  |
| SP_2093 |  |  |  |  |  | *hup, rpoD, rr02, smrC* |  |  |
| SP_1794 |  |  |  |  |  | *hup, rpoD, rr02, smrC* |  |  |
| SP_2217 |  |  |  |  |  | *argR, hup, rpoD, smrC* |  |  |
| SP_0059 |  |  |  |  |  | *argR, codY, hup, rpoD, rr02* |  |  |
| SP_0245 |  |  |  |  |  | *hup, rpoD, rr02, scrR* |  |  |
| SP_0316-SP_0320 |  |  |  |  |  | *argR, codY, hup, rpoD, rr02, scrR* |  |  |
| SP_0321-SP_0327 |  |  |  |  |  | *argR, hup, rpoD, scrR* |  |  |
| SP_0428 |  |  |  |  |  | *hup, rpoD, rr02, scrR* |  |  |
| SP_0431 |  |  |  |  |  | *hup, rpoD, rr02, scrR* |  |  |
| SP_0531 |  |  |  |  |  | *argR, codY, rpoD, rr02* |  |  |
| SP_0645-SP_0647 |  |  |  |  |  | *argR, hup, rpoD, rr02* |  |  |
| SP_0687 |  |  |  |  |  | *hup, rpoD, rr02, scrR* |  |  |
| SP_0759 |  |  |  |  |  | *codY, hup, rpoD, rr02* |  |  |
| SP_1037 |  |  |  |  |  | *codY, hup, rr02, scrR* |  |  |
| SP_1422-SP_1423 |  |  |  |  |  | *argR, hup, rpoD, rr02* |  |  |
| SP_1428 |  |  |  |  |  | *codY, hup, rpoD, rr02* |  |  |
| SP_1432 |  |  |  |  |  | *argR, codY, rpoD, rr02* |  |  |
| SP_1447 |  |  |  |  |  | *hup, rpoD, rr02, scrR* |  |  |
| SP_1528 |  |  |  |  |  | *codY, hup, rpoD, rr02* |  |  |
| SP_1608 |  |  |  |  |  | *argR, codY, hup, rpoD, rr02* |  |  |
| SP_1611 |  |  |  |  |  | *argR, hup, rpoD, rr02* |  |  |
| SP_1800 |  |  |  |  |  | *argR, codY, hup, rr02, scrR* |  |  |
| SP_1995 |  |  |  |  |  | *argR, hup, rpoD, rr02* |  |  |
| SP_2031-2032 |  |  |  |  |  | *hup, rpoD, rr02, scrR* |  |  |
| SP_2147 |  |  |  |  |  | *codY, hup, rpoD, rr02, scrR* |  |  |
| SP_2184-2186 |  |  |  |  |  | *hup, rpoD, rr02, scrR* |  |  |
| SP_2231 |  |  |  |  |  | *argR, hup, rpoD, rr02* |  |  |
| SP_1936 |  |  |  |  |  | *argR, codY, hup, scrR* |  |  |
| SP_1996 |  |  |  |  |  | *argR, codY, hup, rpoD* |  |  |
