# Supplementary material for: A functional genomics catalogue of activated transcription factors during pathogenesis of pneumococcal disease
Source: BMC Genomics. 2014 Sep 8;15(1):769. doi: 10.1186/1471-2164-15-769 (PMC4171566; doi:10.1186/1471-2164-15-769)
Supplement: Supplementary file 10 — Additional file 10: Table S9: Transcription factor-specific upregulated genes shared by S pnemoniae WCH16, WCH43 and D39 during pathogenesis. (DOCX 16 KB) [file 12864_2014_6462_MOESM10_ESM.docx]

**Table S9.** Transcription factor-specific upregulated genes shared by *S pnemoniae* WCH16, WCH43 and D39 during pathogenesis.

| **TF** | **Lungs vsNasopharynx** | | | **Blood vs Lungs** | | | **Brain vs Blood** | |
| --- | --- | --- | --- | --- | --- | --- | --- | --- |
|  | **WCH16** | **WCH43** | **D39** | **WCH16** | **WCH43** | **D39** | **WCH16** | **WCH43** |
| SP_0927 (*smrC*) | SP_0693 | SP_0693 | 0 | 0 | SP_1329 | SP_1329 | SP_0693, SP_0904-SP_0906, SP_0914 | SP_0693, SP_0904-SP_0906, SP_0914 |
| SP_1073 (*rpoD*) | SP_0686SP_0693SP_0698 | SP_0686SP_0693SP_0698 | 0 | 0 | SP_1329 | SP_1329 | SP_0421-SP_0431, SP_0686, SP_0693, SP_0698, SP_0758, SP_0904-SP_0906, SP_0914 | SP_0421-SP_0431, SP_0686, SP_0693, SP_0698, SP_0758, SP_0904-SP_0906, SP_0914 |
| SP_1113 (*hup*) | SP_0686SP_0693 | SP_0686SP_0693 | 0 | 0 | SP_1329 | SP_1329 | SP_0421-SP_0431, SP_0686, SP_0693, SP_0758, SP_0904-SP_0906 | SP_0421-SP_0431, SP_0686, SP_0693, SP_0758, SP_0904-SP_0906 |
| SP_1227 (*rr02*) | SP_0698 | SP_0698 | 0 | 0 | 0 | 0 | SP_0421-SP_0431, SP_0686, SP_0698, SP_0758, SP_0904-SP_0906, SP_0914 | SP_0421-SP_0431, SP_0686, SP_0698, SP_0758, SP_0904-SP_0906, SP_0914 |
| SP_1584 (*codY*) | SP_0693 | SP_0693 | 0 | 0 | SP_1329 | SP_1329 | SP_0693, SP_0758 | SP_0693, SP_0758 |
| SP_1725 (*scrR*) | SP_0686SP_0698 | SP_0686SP_0698 | 0 | 0 | 0 | 0 | SP_0421-SP_0431, SP_0686, SP_0698, SP_0904-SP_0906 | SP_0421-SP_0431, SP_0686, SP_0698, SP_0904-SP_0906 |
| SP_2077 (*argR*) | SP_0698 | SP_0698 | 0 | 0 | 0 | 0 | SP_0698, SP_0758, SP_0914 | SP_0698, SP_0758, SP_0914 |
