# Supplementary material for: A functional genomics catalogue of activated transcription factors during pathogenesis of pneumococcal disease
Source: BMC Genomics. 2014 Sep 8;15(1):769. doi: 10.1186/1471-2164-15-769 (PMC4171566; doi:10.1186/1471-2164-15-769)
Supplement: Supplementary file 11 — Additional file 11: Table S10: S. pneumoniae up-regulated genes under the control of highly activated transcription factors and the number of transcription factor binding sites (TFBs) in their promoter regions. (DOCX 144 KB) [file 12864_2014_6462_MOESM11_ESM.docx]

**Table S10.** *S. pneumoniae* up-regulated genes under the control of highly activated transcription factors and the number of transcription factor binding sites (TFBs) in their promoter regions.

| **Lungs vs Nasopharynx** | | | | | | **Blood vs Lungs** | | | | | | **Brain vs Blood** | | | | | | |
| --- | --- | --- | --- | --- | --- | --- | --- | --- | --- | --- | --- | --- | --- | --- | --- | --- | --- | --- |
| **WCH16** | | **WCH43** | | **D39** | | **WCH16** | | **WCH43** | | **D39** | | **WCH16** | | **WCH43** | | **D39** | | |
| **Gene** | **TFBs*^a^*** | **Gene** | **TFBs** | **Gene** | **TFBs** | **Gene** | **TFBs** | **Gene** | **TFBs** | **Gene** | **TFBs** | **Gene** | **TFBs** | **Gene** | **TFBs** | **Gene** | **TFBs** | |
| SP_0325-SP_0326 | 7 | SP_0686 | 5 | SP_1647 | 6 | SP_1058 | 4 | SP_0263 | 5 | SP_0005 | 6 | SP_0393 | 4 | SP_0325- SP_0326 | 7 | ND*^b^* | ND | |
| SP_0393 | 4 | SP_0693 | 5 |  |  |  |  | SP_1329 | 7 | SP_0059 | 5 | SP_0421- SP_0431 | 5 | SP_0327 | 7 |  |  | |
| SP_0421-SP_0431 | 5 | **SP_0698** | **13** |  |  |  |  | **SP_2182** | **18** | SP_0105 | 6 | SP_0502 | 5 | SP_0421-SP_0431 | 5 |  |  | |
| SP_0686 | 4 |  |  |  |  |  |  |  |  | SP_0116 | 7 | SP_0686 | 4 | SP_0686 | 4 |  |  | |
| SP_0693 | 5 |  |  |  |  |  |  |  |  | **SP_0124- SP_0125** | **23** | SP_0693 | 5 | SP_0693 | 5 |  |  | |
| **SP_0698** | **13** |  |  |  |  |  |  |  |  | SP_0245 | 6 | **SP_0698** | **13** | **SP_0698** | **13** |  |  | |
| SP_0904-SP_0906 | 7 |  |  |  |  |  |  |  |  | SP_0316- SP_0320 | 8 | SP_0758 | 6 | SP_0758 | 6 |  |  | |
| SP_0914 | 5 |  |  |  |  |  |  |  |  | SP_0321- SP_0327 | 7 | SP_0904- SP_0906 | 7 | SP_0904- SP_0906 | 7 |  |  | |
|  |  |  |  |  |  |  |  |  |  | SP_0379- SP_0380 | 5 | SP_0914 | 6 | SP_0914 | 6 |  |  | |
|  |  |  |  |  |  |  |  |  |  | SP_0385 | 4 |  |  | SP_1324 | 9 |  |  | |
|  |  |  |  |  |  |  |  |  |  | SP_0394- SP_0397 | 4 |  |  |  |  |  |  | |
|  |  |  |  |  |  |  |  |  |  | SP_0428 | 5 |  |  |  |  |  |  | |
|  |  |  |  |  |  |  |  |  |  | SP_0431 | 5 |  |  |  |  |  |  | |
|  |  |  |  |  |  |  |  |  |  | SP_0531 | 6 |  |  |  |  |  |  | |
|  |  |  |  |  |  |  |  |  |  | SP_0588- SP_0586 | 4 |  |  |  |  |  |  | |
|  |  |  |  |  |  |  |  |  |  | SP_0645- SP_0647 | 6 |  |  |  |  |  |  | |
|  |  |  |  |  |  |  |  |  |  | SP_0687 | 4 |  |  |  |  |  |  | |
|  |  |  |  |  |  |  |  |  |  | SP_0693 | 5 |  |  |  |  |  |  | |
|  |  |  |  |  |  |  |  |  |  | SP_0714 | 5 |  |  |  |  |  |  | |
|  |  |  |  |  |  |  |  |  |  | SP_0759 | 8 |  |  |  |  |  |  | |
|  |  |  |  |  |  |  |  |  |  | **SP_0874** | **26** |  |  |  |  |  |  | |
|  |  |  |  |  |  |  |  |  |  | SP_0893- SP_0894 | 8 |  |  |  |  |  |  | |
|  |  |  |  |  |  |  |  |  |  | SP_0904- SP_0906 | 7 |  |  |  |  |  |  | |
|  |  |  |  |  |  |  |  |  |  | SP_0907 | 7 |  |  |  |  |  |  | |
|  |  |  |  |  |  |  |  |  |  | **SP_1036** | **25** |  |  |  |  |  |  | |
|  |  |  |  |  |  |  |  |  |  | SP_1037 | 6 |  |  |  |  |  |  | |
|  |  |  |  |  |  |  |  |  |  | SP_1315- SP_1331 | 7 |  |  |  |  |  |  | |
|  |  |  |  |  |  |  |  |  |  | SP_1422- SP_1423 | 8 |  |  |  |  |  |  | |
|  |  |  |  |  |  |  |  |  |  | SP_1428 | 6 |  |  |  |  |  |  | |
|  |  |  |  |  |  |  |  |  |  | SP_1432 | 5 |  |  |  |  |  |  | |
|  |  |  |  |  |  |  |  |  |  | SP_1447 | 6 |  |  |  |  |  |  | |
|  |  |  |  |  |  |  |  |  |  | SP_1528 | 7 |  |  |  |  |  |  | |
|  |  |  |  |  |  |  |  |  |  | **SP_1608** | **13** |  |  |  |  |  |  | |
|  |  |  |  |  |  |  |  |  |  | SP_1611 | 4 |  |  |  |  |  |  | |
|  |  |  |  |  |  |  |  |  |  | SP_1658 | 9 |  |  |  |  |  |  | |
|  |  |  |  |  |  |  |  |  |  | SP_1794 | 5 |  |  |  |  |  |  | |
|  |  |  |  |  |  |  |  |  |  | SP_1800 | 12 |  |  |  |  |  |  | |
|  |  |  |  |  |  |  |  |  |  | SP_1936 | 5 |  |  |  |  |  |  | |
|  |  |  |  |  |  |  |  |  |  | SP_1995- SP_1996 | 5 |  |  |  |  |  |  | |
|  |  |  |  |  |  |  |  |  |  | SP_2031- SP_2032 | 5 |  |  |  |  |  |  | |
|  |  |  |  |  |  |  |  |  |  | SP_2093 | 5 |  |  |  |  |  |  | |
|  |  |  |  |  |  |  |  |  |  | SP_2147 | 7 |  |  |  |  |  |  | |
|  |  |  |  |  |  |  |  |  |  | SP_2184- SP_2186 | 5 |  |  |  |  |  |  | |
|  |  |  |  |  |  |  |  |  |  | SP_2217 | 5 |  |  |  |  |  |  | |
|  |  |  |  |  |  |  |  |  |  | SP_2231 | 6 |  |  |  |  |  |  |  |

*^a^*TFBs: Number of transcription factor binding sites on the promoter region.

*^b^*ND= Not determined.
