# Supplementary material for: A functional genomics catalogue of activated transcription factors during pathogenesis of pneumococcal disease
Source: BMC Genomics. 2014 Sep 8;15(1):769. doi: 10.1186/1471-2164-15-769 (PMC4171566; doi:10.1186/1471-2164-15-769)
Supplement: Supplementary file 12 — Additional file 12: Table S11: Domain activation profiles of S. pneumoniae WCH16, WCH43, and D39 during transition from the nasopharynx → lungs → blood → brain. (DOCX 158 KB) [file 12864_2014_6462_MOESM12_ESM.docx]

**Table S11.** Domain activation profiles of *S. pneumoniae* WCH16, WCH43, and D39 during transition from the nasopharynx→lungs→blood→brain.

**WCH16**

| **Lungs vs Nasopharynx** | **Blood vs Lungs** | **Brain vs Blood** |
| --- | --- | --- |
| ABC_tran (ABC transporter) 2 | N6_N4_Mtase (DNA methyltransferase) | AAA_31 (AAA proteins) |
| Abi (CAAX protease self-immunity) |  | ABC_tran (ABC transporter) 2 |
| ACT_5 (ACT domain) |  | Abi (CAAX protease self-immunity) |
| adh_short (Short-chain dehydrogenase) 2 |  | Acetyltransf_1 (Acetyltransferase) |
| Alpha-amylase (Alpha-Amylase) |  | ACT_5 (ACT domain) |
| ALS_ss_C (Small subunit of acetolactate synthase) |  | adh_short(Short-chain dehydrogenase) 2 |
| Amidase (Amidase) |  | Alpha-amylase (Alpha-Amylase) |
| ASCH (ASCH domain) |  | ALS_ss_C (Small subunit of acetolactate synthase) |
| ATP-cone (ATP cone) |  | Amidase (Amidase) |
| BPD_transp_1 (Binding-protein-dependent transport system inner membrane component) |  | ASCH (ASCH domain) |
| CN_hydrolase (Carbon-nitrogen hydrolase) |  | ATP-cone(ATP cone) |
| DDE_Tnp_1_4 (Transposase DDE domain group 1) |  | Bac_transf (Bacterial sugar transferase) |
|  |  |  |
| DUF1430 (Protein of unknown function) |  | Biotin_lipoyl (Biotin attachment domain) |
| DUF1980 (Domain of unknown function) |  | BPD_transp_2 (Branched-chain amino acid transport system / permease component) |
| DUF2128 (Uncharacterized protein conserved in bacteria) |  | CN_hydrolase (Carbon-nitrogen hydrolase) |
| DUF2129 (Uncharacterized protein conserved in bacteria) |  | DDE_3 (DDE superfamily endonuclease) |
| DUF318 (Predicted permease) |  | DDE_Tnp_1_4 (Transposase DDE domain group 1) |
| DUF3358 (Domain of unknown function) |  | DegV (Uncharacterised protein, DegV family COG1307) |
| DUF910 (Bacterial protein of unknown function) |  | DHO_dh (Dihydroorotate dehydrogenase) |
| EFP (Elongation factor P) |  | DNA_pol3_delta (DNA polymerase III holoenzyme) |
| EFP_N (Elongation factor P) |  | DUF1430 (Protein of unknown function) |
| EIID-AGA (PTS system mannose/fructose/sorbose family IID component) |  | DUF1846 (Domain of unknown function) |
| Elong-fact-P_C (Elongation factor P) |  | DUF1980 (Domain of unknown function) |
| FabA (FabA-like domain) |  | DUF2128 (Uncharacterized protein conserved in bacteria) |
| FtsX (FtsX-like permease family) 2 |  | DUF2129 (Uncharacterized protein conserved in bacteria) |
| Glu-tRNAGln (Glu-tRNAGln amidotransferase C subunit) |  | DUF318 (Predicted permease) |
| GTP_EFTU (GTP-binding elongation factor family, EF-Tu/EF-1A subfamily) |  | DUF3358 (Domain of unknown function) |
| GTP_EFTU_D2 (GTP-binding elongation factor family, EF-Tu/EF-1A subfamily) |  | DUF910 (Bacterial protein of unknown function) |
| HTH_1 (Helix-turn-helix) 2 |  | EcoEI_R_C (EcoEI R protein C-terminal domain) |
| IlvC (Acetohydroxy acid isomeroreductase, catalytic domain) |  | F_bP_aldolase (Fructose-bisphosphate aldolase) |
| IlvN (Acetohydroxy acid isomeroreductase, catalytic domain) |  | FabA (FabA-like domain) |
| ketoacyl-synt (Beta-ketoacyl synthase, N-terminal domain) |  | FtsX (FtsX-like permease family) 3 |
| Ketoacyl-synt_C (Beta-ketoacyl-ACP synthase) |  | Gln-synt_C (Glutamine synthetase) |
| Lactamase_B (Metallo-beta-lactamase protein fold) |  | Gln-synt_N (Glutamine synthetase) |
| LysR_substrate (LysR substrate binding domain) 2 |  | Glu-tRNAGln (Glu-tRNAGln amidotransferase C subunit) |
| MacB_PCD (MacB-like periplasmic core domain) |  | Glycos_transf_1 (Glycosyl transferases group 1) |
| MerR_1 (MerR HTH family regulatory protein) |  | GTP_EFTU (GTP-binding elongation factor family, EF-Tu/EF-1A subfamily) |
| Na_H_Exchanger (Sodium/hydrogen exchanger family) |  | GTP_EFTU_D2 (GTP-binding elongation factor family, EF-Tu/EF-1A subfamily) |
| OMPdecase (Orotidine 5'-phosphate decarboxylase / HUMPS family) |  | HI0933_like (HI0933-like protein) |
| Orn_DAP_Arg_deC (Group IV pyridoxal-dependent decarboxylases) |  | HTH_1 (Helix-turn-helix) 2 |
| PAD_porph (Porphyromonas-type peptidyl-arginine deiminase) |  | HTH_3 (Helix-turn-helix) |
| PadR (Transcriptional regulator PadR-like family) |  | IlvC (Acetohydroxy acid isomeroreductase, catalytic domain) |
| PASTA (PASTA domain) |  | IlvN (Acetohydroxy acid isomeroreductase, catalytic domain) |
| PBP_dimer (Penicillin-binding Protein dimerisation domain) |  | ketoacyl-synt (Beta-ketoacyl synthase, N-terminal domain) |
| Peptidase_A8 (Signal peptidase (SPase) II) |  | Ketoacyl-synt_C (Beta-ketoacyl-ACP synthase) |
| Peptidase_M1 (Peptidase family M1) |  | KH_1 (KH domain) |
| Peptidase_M50 (Peptidase family M50) |  | Lactamase_B (Metallo-beta-lactamase protein fold) |
| PEP-utilizers_C (PEP-utilising enzyme, TIM barrel domain) |  | LysR_substrate (LysR substrate binding domain) 2 |
| Pribosyltran (Phosphoribosyl transferase domain) |  | MacB_PCD (MacB-like periplasmic core domain) |
| Pro_isomerase (Cyclophilin type peptidyl-prolyl cis-trans isomerase/CLD) |  | MerR_1 (MerR HTH family regulatory protein) |
| PseudoU_synth_2 (RNA pseudouridylate synthase) |  | Methyltransf_5 (MraW methylase family) |
| RelA_SpoT (Region found in RelA / SpoT proteins) |  | NAD_binding_1 (Oxidoreductase NAD-binding domain) |
| Response_reg (Response regulator receiver domain) |  | NUDIX (Nudix family) |
| Rhodanese (Rhodanese) |  | OMPdecase (Orotidine 5'-phosphate decarboxylase / HUMPS family) |
| RICH (RICH domain) |  | Orn_DAP_Arg_deC (Group IV pyridoxal-dependent decarboxylases) |
| S4 (S4 domain) |  | PAD_porph (Porphyromonas-type peptidyl-arginine deiminase) |
| Saccharop_dh (Saccharopine dehydrogenase) |  | PadR (Transcriptional regulator PadR-like family) |
| TipAS (TipAS antibiotic-recognition domain) |  | PASTA (PASTA domain) |
| TPP_enzyme_C (Thiamine pyrophosphate enzyme, C-terminal TPP binding domain) |  | PBP_dimer (Penicillin-binding Protein dimerisation domain) |
| TPP_enzyme_M (Thiamine pyrophosphate enzyme, central domain) |  | PCRF (PCRF domain) |
| TPP_enzyme_N (Thiamine pyrophosphate enzyme, N-terminal TPP binding domain) |  | Peptidase_A8 (Signal peptidase (SPase) II) |
| Trans_reg_C (Transcriptional regulatory protein, C terminal) |  | Peptidase_M1 (Peptidase family M1) |
| Transpeptidase (Penicillin binding proteins) |  | Peptidase_M50 (Peptidase family M50) |
| tRNA_bind (Putative tRNA binding domain) |  | PEP-utilizers_C (PEP-utilising enzyme, TIM barrel domain) |
| tRNA_m1G_MT (tRNA (Guanine-1)-methyltransferase) |  | Peripla_BP_6 (Periplasmic binding protein) |
| tRNA-synt_1g (tRNA synthetases class I (M)) |  | Phe_tRNA-synt_N (Aminoacyl tRNA synthetase class II, N-terminal domain) |
| Virul_fac_BrkB (Virulence factor BrkB) |  | PNPase (Polyribonucleotide nucleotidyltransferase, RNA binding domain) |
| YajC (Preprotein translocase subunit) |  | Pribosyltran (Phosphoribosyl transferase domain) |
| YSIRK_signal (YSIRK type signal peptide) |  | Pro_isomerase (Cyclophilin type peptidyl-prolyl cis-trans isomerase/CLD) |
|  |  | PseudoU_synth_2 (RNA pseudouridylate synthase) |
|  |  | PTS_EIIA_1 (phosphoenolpyruvate-dependent sugar phosphotransferase system, EIIA 1) |
|  |  | PTS_EIIB (phosphotransferase system, EIIB) |
|  |  | PTS_EIIC (Phosphotransferase system, EIIC) |
|  |  | ResIII (Type III restriction enzyme, res subunit) |
|  |  | Response_reg (Response regulator receiver domain) 2 |
|  |  | RF-1 (RF-1 domain) |
|  |  | Rhodanese (Rhodanese) |
|  |  | RNase_PH (3' exoribonuclease family, domain 1) 2 |
|  |  | RNase_PH_C (3' exoribonuclease family, domain 2) 2 |
|  |  | ROK (ROK family) |
|  |  | S1 (S1 domain) |
|  |  | S4 (S4 domain) |
|  |  | Saccharop_dh (Saccharopine dehydrogenase) |
|  |  | Sod_Fe_C (Superoxide dismutase) |
|  |  | Sod_Fe_N (Superoxide dismutase) |
|  |  | TipAS (TipAS antibiotic-recognition domain) |
|  |  | TPP_enzyme_C (Thiamine pyrophosphate enzyme, C-terminal TPP binding domain) |
|  |  | TPP_enzyme_M (Thiamine pyrophosphate enzyme, central domain) |
|  |  | TPP_enzyme_N (Thiamine pyrophosphate enzyme, N-terminal TPP binding domain) |
|  |  | Trans_reg_C (Transcriptional regulatory protein, C terminal) 2 |
|  |  | Transpeptidase (Penicillin binding proteins) |
|  |  | tRNA_bind (Putative tRNA binding domain) |
|  |  | tRNA_m1G_MT (tRNA (Guanine-1)-methyltransferase) |
|  |  | tRNA-synt_1g (tRNA synthetases class I (M)) |
|  |  | tRNA-synt_2d (tRNA synthetases class II core domain (F)) |

**WCH43**

| **Lungs vs Nasopharynx** | **Blood vs Lungs** | **Brain vs Blood** |
| --- | --- | --- |
| adh_short(Short-chain dehydrogenase) | ADP_ribosyl_GH (ADP-ribosylglycohydrolase) | AAA_31 (AAA proteins) |
| ASCH (ASCH domain) | BPD_transp_1 (Binding-protein-dependent transport system inner membrane component) | ABC_tran (ABC transporter) 2 |
| ATP-cone(ATP cone) |  | Abi (CAAX protease self-immunity) |
| DUF1430(Protein of unknown function) | ComC (COMC family) | Acetyltransf_1 (Acetyltransferase) 2 |
| DUF2128 (Uncharacterized protein conserved in bacteria) | DAGK_cat (Diacylglycerol kinase) | ACT_5 (ACT domain) |
| DUF3358 (Domain of unknown function) | DDE_4 (DDE superfamily endonuclease) | adh_short(Short-chain dehydrogenase) 2 |
| DUF910(Bacterial protein of unknown function) | DHDPS (Dihydrodipicolinate synthase) | Alpha-amylase (Alpha-Amylase) |
| FtsX (FtsX-like permease family) | GreA_GreB (Transcription elongation factor, GreA/GreB, C-term) | ALS_ss_C (Small subunit of acetolactate synthase) |
| HTH_1 (Helix-turn-helix) |  | Amidase (Amidase) |
| LysR_substrate(LysR substrate binding domain) | GreA_GreB_N (Transcription elongation factor, N-terminal) | ASCH (ASCH domain) |
| MacB_PCD (MacB-like periplasmic core domain) | MS_channel (Mechanosensitive ion channel) | ATP-cone (ATP cone) |
| PadR (Transcriptional regulator PadR-like family) | OpuAC (Substrate binding domain of ABC-type glycine betaine transport system) | ATP-grasp_4 (ATP-grasp domain) |
| Peptidase_M1 (Peptidase family M1) |  | B3_4 (B3/4 domain) |
| Peptidase_M50 (Peptidase family M50) | PBP_dimer (Penicillin-binding Protein dimerisation domain) | B5 (tRNA synthetase B5 domain) |
| PEP-utilizers_C (PEP-utilising enzyme, TIM barrel domain) |  | Bac_transf (Bacterial sugar transferase) |
| Pribosyltran (Phosphoribosyl transferase domain) | Peptidase_M50 (Peptidase family M50) | Biotin_lipoyl (Biotin attachment domain) |
| Pro_isomerase (Cyclophilin type peptidyl-prolyl cis-trans isomerase/CLD) | Ribosomal_L23 (Ribosomal protein L23) | BPD_transp_2 (Branched-chain amino acid transport system / permease component) |
| Response_reg (Response regulator receiver domain) | Transpeptidase (Penicillin binding proteins) | CN_hydrolase (Carbon-nitrogen hydrolase) |
| Rhodanese (Rhodanese) |  | DDE_3 (DDE superfamily endonuclease) |
| Trans_reg_C (Transcriptional regulatory protein, C terminal) |  | DDE_Tnp_1_4 (Transposase DDE domain group 1) |
| tRNA_bind (Putative tRNA binding domain) |  | DegV (Uncharacterised protein, DegV family COG1307) |
| tRNA_m1G_MT (tRNA (Guanine-1)-methyltransferase) |  | DHO_dh (Dihydroorotate dehydrogenase) |
| tRNA-synt_1g (tRNA synthetases class I (M)) |  | DNA_pol3_delta (DNA polymerase III holoenzyme) |
|  |  | DUF1430 (Protein of unknown function) |
|  |  | DUF1846 (Domain of unknown function) |
|  |  | DUF1980 (Domain of unknown function) |
|  |  | DUF2128 (Uncharacterized protein conserved in bacteria) |
|  |  | DUF2129 (Uncharacterized protein conserved in bacteria) |
|  |  | DUF318 (Predicted permease) |
|  |  | DUF3358 (Domain of unknown function) |
|  |  | DUF910 (Bacterial protein of unknown function) |
|  |  | EIID-AGA (PTS system mannose/fructose/sorbose family IID component) |
|  |  | Exo_endo_phos (Endonuclease/Exonuclease/phosphatase family) |
|  |  | F_bP_aldolase (Fructose-bisphosphate aldolase) |
|  |  | FabA (FabA-like domain) |
|  |  | FDX-ACB (Phenylalanine-tRNA ligase) |
|  |  | Fer4_13 (4Fe-4S single cluster domain) |
|  |  | FtsX (FtsX-like permease family) 3 |
|  |  | Glu-tRNAGln (Glu-tRNAGln amidotransferase C subunit) |
|  |  | Glycos_transf_1 (Glycosyl transferases group 1) |
|  |  | GTP_EFTU (GTP-binding elongation factor family, EF-Tu/EF-1A subfamily) |
|  |  | GTP_EFTU_D2 (GTP-binding elongation factor family, EF-Tu/EF-1A subfamily) |
|  |  | HAMP (HAMP domain) |
|  |  | Hepar_II_III (Heparinase II/III-like protein) |
|  |  | HI0933_like (HI0933-like protein) |
|  |  | HisKA (Two-component regulatory system) |
|  |  | HTH_1 (Helix-turn-helix) 2 |
|  |  | HTH_3 (Helix-turn-helix) |
|  |  | IlvC (Acetohydroxy acid isomeroreductase, catalytic domain) |
|  |  | IlvN (Acetohydroxy acid isomeroreductase, catalytic domain) |
|  |  | ketoacyl-synt (Beta-ketoacyl synthase, N-terminal domain) |
|  |  | Ketoacyl-synt_C (Beta-ketoacyl-ACP synthase) |
|  |  | Lactamase_B (Metallo-beta-lactamase protein fold) |
|  |  | LysR_substrate (LysR substrate binding domain) 2 |
|  |  | MacB_PCD (MacB-like periplasmic core domain) |
|  |  | MerR_1 (MerR HTH family regulatory protein) |
|  |  | Methyltransf_5 (MraW methylase family) |
|  |  | NAD_binding_1 (Oxidoreductase NAD-binding domain) |
|  |  | NUDIX (Nudix family) |
|  |  | OMPdecase (Orotidine 5'-phosphate decarboxylase / HUMPS family) |
|  |  | Orn_DAP_Arg_deC (Group IV pyridoxal-dependent decarboxylases) |
|  |  | PAD_porph (Porphyromonas-type peptidyl-arginine deiminase) |
|  |  | PadR (Transcriptional regulator PadR-like family) |
|  |  | PASTA (PASTA domain) |
|  |  | PBP_dimer (Penicillin-binding Protein dimerisation domain) |
|  |  | PCRF (PCRF domain) |
|  |  | Peptidase_A8 (Signal peptidase (SPase) II) |
|  |  | Peptidase_M1 (Peptidase family M1) |
|  |  | Peptidase_M50 (Peptidase family M50) |
|  |  | PEP-utilizers_C (PEP-utilising enzyme, TIM barrel domain) |
|  |  | Peripla_BP_6 (Periplasmic binding protein) |
|  |  | Phage_integrase (Phage integrase family) |
|  |  | Phe_tRNA-synt_N (Aminoacyl tRNA synthetase class II, N-terminal domain) |
|  |  | Pro_isomerase (Cyclophilin type peptidyl-prolyl cis-trans isomerase/CLD) |
|  |  | PseudoU_synth_2 (RNA pseudouridylate synthase) |
|  |  | PTS_EIIA_1 (phosphoenolpyruvate-dependent sugar phosphotransferase system, EIIA 1) |
|  |  | PTS_EIIB (phosphotransferase system, EIIB) |
|  |  | PTS_EIIC (Phosphotransferase system, EIIC) |
|  |  | Response_reg (Response regulator receiver domain) 2 |
|  |  | RF-1 (RF-1 domain) |
|  |  | Rhodanese (Rhodanese) |
|  |  | Ribosomal_L6 (Ribosomal protein L6) 2 |
|  |  | ROK (ROK family) |
|  |  | S4 (S4 domain) |
|  |  | Saccharop_dh (Saccharopine dehydrogenase) |
|  |  | Sod_Fe_C (Superoxide dismutase) |
|  |  | Sod_Fe_N (Superoxide dismutase) |
|  |  | TipAS (TipAS antibiotic-recognition domain) |
|  |  | TPP_enzyme_C (Thiamine pyrophosphate enzyme, C-terminal TPP binding domain) |
|  |  | TPP_enzyme_M (Thiamine pyrophosphate enzyme, central domain) |
|  |  | TPP_enzyme_N (Thiamine pyrophosphate enzyme, N-terminal TPP binding domain) |
|  |  | Trans_reg_C (Transcriptional regulatory protein, C terminal) 2 |
|  |  | Transpeptidase (Penicillin binding proteins) |
|  |  | tRNA_bind (Putative tRNA binding domain) 2 |
|  |  | tRNA_m1G_MT (tRNA (Guanine-1)-methyltransferase) |
|  |  | tRNA-synt_1g (tRNA synthetases class I (M)) |
|  |  | tRNA-synt_2d (tRNA synthetases class II core domain (F)) |
|  |  | YajC (Preprotein translocase subunit) |

**D39**

| **Lungs vs Nasopharynx** | **Blood vs Lungs** | |
| --- | --- | --- |
| ABC_tran (ABC transporter) 3 | 2H-phosphodiest (Domain of unknown function (DUF1868)) | IMS (impB/mucB/samB family) |
| Bac_transf (Bacterial sugar transferase) | 3H (3H domain) | IMS_C (impB/mucB/samB family C-terminal) |
| Bmp (Basic membrane protein) | AAA (ATPase family associated with various cellular activities (AAA)) | IMS_HHH (IMS family HHH motif) |
| DegV (Uncharacterised protein, DegV family COG1307) | AAA_14 (AAA protein) | IstB_IS21 (IstB-like ATP binding protein) |
| DnaI_N (Primosomal protein DnaI N-terminus) | AAA_18 (AAA proteins) | KH_4 (KH domain) |
| EPSP_synthase (EPSP synthase) | AAA_2 (AAA proteins) | LacAB_rpiB (Ribose/Galactose Isomerase) |
| FAD_syn (Prokaryotic riboflavin biosynthesis protein) | AAA_21 (AAA proteins) | LacI (Bacterial regulatory proteins, lacI family) |
| Flavokinase (Riboflavin kinase) | AAA_31 (AAA proteins) | Lactamase_B_2 (Beta-lactamase superfamily domain) |
| FtsX (FtsX-like permease family) | ABC_cobalt (ABC-type cobalt transport system, permease component) | Lactococcin_972 (Bacteriocin) |
| G6PD_C (Glucose-6-phosphate dehydrogenase) | ABC_membrane (Transmembrane domain of ABC transporters) | LANC_like (Lanthionine synthetase C-like protein) |
| G6PD_N (Glucose-6-phosphate dehydrogenase) | ABC_tran (ABC transporter) 13 | LysR_substrate (LysR substrate binding domain) |
| HD (HD domain) | ABC2_membrane (ABC transporter) | LytTR (LytTr DNA-binding domain) |
| IstB_IS21 (IstB-like ATP binding protein) | ABC2_membrane_3 (ATP-binding cassette transporter) | Mac (Maltose acetyltransferase) |
| M20_dimer (Peptidase dimerisation domain) | Abhydrolase_6 (Alpha/beta hydrolase fold) | MacB_PCD (MacB-like periplasmic core domain) |
|  |  | Mannitol_dh (Mannitol dehydrogenase Rossmann domain) |
| MatE (Multi antimicrobial extrusion protein) 2 | Abi (CAAX protease self-immunity) 7 | Mannitol_dh_C (Mannitol dehydrogenase C-terminal domain) |
| Mur_ligase_C (Mur ligase family, glutamate ligase domain) | ABM (ABM domain) | MarR_2 (MarR family) |
| Mur_ligase_M (Mur ligase middle domain) | Acetyltransf_1 (Acetyltransferase) | McrBC (McrBC 5-methylcytosine restriction system component) |
| Peptidase_M13 (Peptidase family M13) | Acetyltransf_3 (Acetyltransferase) 2 | MerR_1 (MerR HTH family regulatory protein) |
| Peptidase_M13_N (Peptidase family M13) | Adenine_glyco (Methyladenine glycosylase) | Metallophos_2 (Calcineurin-like phosphoesterase superfamily domain) |
| Peptidase_M20 (Peptidase family M20/M25/M40) | adh_short (Short-chain dehydrogenase) | Meth_synt_1 (Cobalamin-independent synthase, N-terminal domain) |
| PGI (Glucose-6-phosphate isomerase) | Aldedh (Aldehyde dehydrogenase family) | Meth_synt_2 (Cobalamin-independent synthase, Catalytic domain) |
| RNA_pol_A_bac (RNA polymerase Rpb3/RpoA insert domain) | Aldolase (KDPG and KHG aldolase) | Methylase_S (Type I restriction modification DNA specificity domain) |
| RNA_pol_A_CTD (Bacterial RNA polymerase, alpha chain C terminal domain) | Aldolase_II (Class II Aldolase and Adducin N-terminal domain) 2 | Methyltransf_5 (MraW methylase family) |
| RNA_pol_L (RNA polymerase Rpb3/Rpb11 dimerisation domain) | Alpha-amylase (Alpha-Amylase) 3 | MFS_1 (Major facilitator family) 2 |
| SMC_N (RecF/RecN/SMC N terminal domain) | Alpha-amylase_C (Alpha-Amylase) | MFS_3 (Domain of unknown function) |
| Transket_pyr (Transketolase, pyrimidine binding domain) | Anth_synt_I_N (Anthranilate synthase component I, N terminal region) | Mga (Mga helix-turn-helix domain) |
| Transketolase_C (Transketolase, C-terminal domain) | AP_endonuc_2 (Xylose isomerase-like TIM barrel) | MgtC (MgtC family) |
| Transketolase_N (Transketolase, thiamine diphosphate binding domain) | Arg_repressor (Arginine repressor ArgR) | MIP (Major intrinsic proteins) |
| ABC_tran (ABC transporter) 3 | Arg_repressor_C (Arginine repressor ArgR) | MMR_HSR1 (50S ribosome-binding GTPase) |
| Bac_transf (Bacterial sugar transferase) | ATP-grasp_4 (ATP-grasp domain) | MobC (Bacterial mobilisation protein (MobC)) |
| Bmp (Basic membrane protein) | ATP-synt_ab (ATP synthase alpha/beta subunits) 2 | MreD (rod shape-determining protein MreD) |
| DegV (Uncharacterised protein, DegV family COG1307) | ATP-synt_ab_C (ATP synthase alpha/beta subunits) 2 | MTHFR (Methylenetetrahydrofolate reductase) |
| DnaI_N (Primosomal protein DnaI N-terminus) | ATP-synt_ab_N (ATP synthase alpha/beta subunits) 2 | MTS (Methyltransferase small domain) |
| EPSP_synthase (EPSP synthase) | ATP-synt_C (ATP synthase subunit C) 2 | MucBP (MucBP domain) |
| FAD_syn (Prokaryotic riboflavin biosynthesis protein) | ATP-synt_D (ATP synthase subunit D) | Mur_ligase_M (Mur ligase middle domain) |
| Flavokinase (Riboflavin kinase) | ATP-synt_F (ATP6V1F) | N6_Mtase (N-6 DNA Methylase) |
| FtsX (FtsX-like permease family) | AzlD (Branched-chain amino acid transport protein (AzlD)) | Na_Ala_symp (Sodium:alanine symporter family) |
| G6PD_C (Glucose-6-phosphate dehydrogenase) | Bac_luciferase (Luciferase) | NAD_binding_10 (NADH(P)-binding) |
| G6PD_N (Glucose-6-phosphate dehydrogenase) | Bac_transf (Bacterial sugar transferase) | NanE (Putative N-acetylmannosamine-6-phosphate epimerase) |
| HD (HD domain) | Bacteriocin_IIc (Bacteriocin class II with double-glycine leader peptide) 5 | NUDIX (Nudix family) |
| IstB_IS21 (IstB-like ATP binding protein) | BioY (BioY family) | O-ag_pol_Wzy (O-antigen polysaccharide polymerase Wzy) |
| M20_dimer (Peptidase dimerisation domain) | BNR_2 (Bacterial neuraminidase) | OMPdecase (Orotidine 5'-phosphate decarboxylase / HUMPS family) 2 |
|  | BPD_transp_1 (Binding-protein-dependent transport system inner membrane component) 17 | Orn_Arg_deC_N (Group IV pyridoxal-dependent decarboxylases) |
| MatE (Multi antimicrobial extrusion protein) 2 | CAT_RBD (CAT RNA-binding domain) 2 | Orn_DAP_Arg_deC (Group IV pyridoxal-dependent decarboxylases) |
| Mur_ligase_C (Mur ligase family, glutamate ligase domain) | CbiQ (Cobalamin biosynthesis) | PadR (Transcriptional regulator PadR-like family) |
| Mur_ligase_M (Mur ligase middle domain) | CBM_48 (Carbohydrate-binding module) | PALP (Pyridoxal-phosphate dependent enzyme) |
| Peptidase_M13 (Peptidase family M13) | CBS (CBS domain) 2 | PAP2 (PAP2 superfamily) 2 |
| Peptidase_M13_N (Peptidase family M13) | Chorismate_bind (chorismate binding enzyme) | PBP_like_2 (PBP superfamily domain) 2 |
| Peptidase_M20 (Peptidase family M20/M25/M40) | ClpB_D2-small (C-terminal, D2-small domain, of ClpB protein) | Pectate_lyase_3 (Pectate lyase superfamily protein) |
| PGI (Glucose-6-phosphate isomerase) | CN_hydrolase (Carbon-nitrogen hydrolase) | Pept_tRNA_hydro (Peptidyl-tRNA hydrolase) |
| RNA_pol_A_bac (RNA polymerase Rpb3/RpoA insert domain) | Cna_B (Cna protein B-type domain) 5 | Peptidase_M22 (Glycoprotease family) |
| RNA_pol_A_CTD (Bacterial RNA polymerase, alpha chain C terminal domain) | CoA_binding_2 (CoA binding domain) | Peptidase_M26_C (M26 IgA1-specific Metallo-endopeptidase C-terminal region) |
| RNA_pol_L (RNA polymerase Rpb3/Rpb11 dimerisation domain) | CoiA (Competence protein CoiA-like family) | Peptidase_M26_N (M26 IgA1-specific Metallo-endopeptidase N-terminal region) |
| SMC_N (RecF/RecN/SMC N terminal domain) | ComC (COMC family) | Peptidase_M50 (Peptidase family M50) |
| Transket_pyr (Transketolase, pyrimidine binding domain) | DAGK_prokar (Diacylglycerol kinase) | Peptidase_S15 (X-Pro dipeptidyl-peptidase (S15 family)) |
| Transketolase_C (Transketolase, C-terminal domain) | DAO (FAD dependent oxidoreductase family) 2 | Peptidase_S66 (LD-carboxypeptidase) |
| Transketolase_N (Transketolase, thiamine diphosphate binding domain) | dCMP_cyt_deam_1 (Cytidine and deoxycytidylate deaminase zinc-binding region) 2 | PepX_C (X-Pro dipeptidyl-peptidase C-terminal non-catalytic domain) |
|  | DDE_3 (DDE superfamily endonuclease) 2 | PepX_N (X-Prolyl dipeptidyl aminopeptidase PepX, N-terminal) |
|  | DDE_Tnp_1_4 (Transposase DDE domain group 1) | Peripla_BP_3 (Periplasmic binding protein-like domain) |
|  | DDE_Tnp_1_6 (Transposase DDE domain) | PfkB (pfkB family carbohydrate kinase) 2 |
|  | DDE_Tnp_ISL3 (Transposase) | PFL (Pyruvate formate lyase) |
|  | DeoRC (DeoR C terminal sensor domain) | Phage_integr_N2 (Phage integrase, N-terminal SAM-like domain) |
|  | DHDPS (Dihydrodipicolinate synthase) 2 | Phage_integrase (Phage integrase family) 3 |
|  | DiS_P_DiS (Bacterial Peptidase A24 N-terminal domain) | Phage_pRha (Phage regulatory protein Rha (Phage_pRha)) |
|  | DNA_methylase (DNA methyltransferase) 2 | PhdYeFM_antitox (Antitoxin Phd_YefM, type II toxin-antitoxin system) |
|  | DnaB_2 (Replication initiation and membrane attachment) | Phe_tRNA-synt_N (Aminoacyl tRNA synthetase class II, N-terminal domain) |
|  | DUF1237 (Protein of unknown function) | PhoU (PhoU domain) 2 |
|  | DUF1292 (Protein of unknown function) | PIG-L (GlcNAc-PI de-N-acetylase) |
|  | DUF1294 (Protein of unknown function) | Plasmid_Txe (Toxin-antitoxin system) |
|  | DUF1792 (Domain of unknown function) | PNP_UDP_1 (Phosphorylase superfamily) |
|  | DUF1919 (Domain of unknown function) | Polysacc_syn_2C (Polysaccharide biosynthesis protein C-terminal) |
|  | DUF1975 (Domain of unknown function) 3 | Polysacc_synt (Polysaccharide biosynthesis protein) |
|  | DUF1980 (Domain of unknown function) | Polysacc_synt_2 (Polysaccharide biosynthesis protein) |
|  | DUF2154 (Cell wall-active antibiotics response protein) | PQ-loop (PQ loop repeat) |
|  | DUF2200 (Uncharacterized protein conserved in bacteria) | PRD (PRD domain) 8 |
|  | DUF2812 (Protein of unknown function) | PRD_Mga (M protein trans-acting positive regulator (MGA) PRD domain) 2 |
|  | DUF2829 (Protein of unknown function) | PRiA4_ORF3 (Plasmid pRiA4b ORF-3-like protein) |
|  | DUF3021 (Protein of unknown function) | Pribosyltran (Phosphoribosyl transferase domain) 3 |
|  | DUF3278 (Protein of unknown function) | Pribosyltran_N (N-terminal domain of ribose phosphate pyrophosphokinase) |
|  | DUF3397 (Protein of unknown function) | PS_pyruv_trans (Polysaccharide pyruvyl transferase) |
|  | DUF3502 (Domain of unknown function) | PseudoU_synth_2 (RNA pseudouridylate synthase) |
|  | DUF3666 (Ribose-5-phosphate isomerase) | Pterin_bind (Dihydropteroate synthase) |
|  | DUF386 (Domain of unknown function) 2 | PTS_EIIA_1 (phosphoenolpyruvate-dependent sugar phosphotransferase system, EIIA 1) 2 |
|  | DUF3884 (Protein of unknown function) | PTS_EIIA_2 (Phosphoenolpyruvate-dependent sugar phosphotransferase system, EIIA 2) 7 |
|  | DUF4300 (Domain of unknown function) | PTS_EIIB (phosphotransferase system, EIIB) 2 |
|  | DUF624 (Protein of unknown function, DUF624) | PTS_EIIC (Phosphotransferase system, EIIC) 8 |
|  | DUF77 (Domain of unknown function DUF77) | PTS_IIA (PTS system, Lactose/Cellobiose specific IIA subunit) 3 |
|  | EamA (EamA-like transporter family) 2 | PTS_IIB (PTS system, Lactose/Cellobiose specific IIB subunit) 8 |
|  | EIIA-man (PTS system fructose IIA component) 3 | PTSIIB_sorb (PTS system sorbose subfamily IIB component) 2 |
|  | EIIC-GAT (PTS system sugar-specific permease component) 2 | PulG (Type II secretory pathway pseudopilin) |
|  | EIID-AGA (PTS system mannose/fructose/sorbose family IID component) 2 | QueT (QueT transporter) |
|  | EII-Sor (PTS system sorbose-specific iic component) | RadC (Domain of unknown function) |
|  | Epimerase (NAD dependent epimerase/dehydratase family) 3 | Radical_SAM (Radical SAM superfamily) |
|  | Epimerase_2 (UDP-N-acetylglucosamine 2-epimerase) 2 | RBFA (Ribosome-binding factor A) |
|  | Epimerase_Csubm(UDP-glucose 4-epimerase C-term subunit) | RbsD_FucU (RbsD / FucU transport protein family) 2 |
|  | Esterase (Putative esterase) 2 | RecX (RecX family) |
|  | ExsB (ExsB) | Redoxin (Redoxin) |
|  | F5_F8_type_C (Discoidin domain) 3 | Relaxase (Relaxase/Mobilisation nuclease domain) |
|  | FA_synthesis (Fatty acid synthesis protein) | RepA_N (Replication initiator protein A (RepA) N-terminus) |
|  | Fer4_12 (4Fe-4S single cluster domain) | ResIII (Type III restriction enzyme, res subunit) |
|  | FGGY_C (FGGY carbohydrate kinase family) | Ribosomal_L33 (Ribosomal protein L33) |
|  | FGGY_N (FGGY carbohydrate kinase family) | Ribul_P_3_epim (Ribulose-phosphate 3-epimerase) |
|  | Fic (Fic/DOC protein family) | RICH (RICH domain) |
|  | Flavoprotein (Flavoprotein) | ROK (ROK family) 3 |
|  | Fst_toxin (Par stability determinant) | RuvA_N (RuvA N terminal domain) |
|  | Ftsk_gamma (Ftsk gamma domain) | S1 (S1 domain) |
|  | FtsK_SpoIIIE (FtsK/SpoIIIE family) | SAM_adeno_trans (S-adenosyl-l-methionine hydroxide adenosyltransferase) |
|  | FtsX (FtsX-like permease family) 2 | SAP (SAP domain) |
|  | **Fucose_iso_C (L-fucose isomerase)** | SBP_bac_1 (Bacterial extracellular solute-binding protein) 2 |
|  | Fucose_iso_N1 (L-fucose isomerase) | SBP_bac_6 (Bacterial extracellular solute-binding protein) |
|  | Fucose_iso_N2 (L-fucose isomerase) | SBP_bac_8 (Bacterial extracellular solute-binding protein) |
|  | G5 (G5 domain) | SDH_alpha (Serine dehydratase alpha chain) |
|  | GalP_UDP_tr_C (Galactose-1-phosphate uridylyltransferase) 2 | SecA_DEAD (SecA DEAD-like domain) |
|  | GalP_UDP_transf (Galactose-1-phosphate uridylyltransferase) 2 | SecA_PP_bind (SecA preprotein cross-linking domain) |
|  | GATase (Glutamine amidotransferase) | SecA_SW (SecA Wing and Scaffold domain) |
|  | GFO_IDH_MocA (Oxidoreductase family, NAD-binding Rossmann fold) 2 | SecY (SecY protein) |
|  | GFO_IDH_MocA_C (Oxidoreductase family, C-terminal alpha/beta domain) 2 | Sialidase (Glycoside hydrolase family 33) |
|  | Gly_kinase (Glycerate kinase) | SIS (SIS domain) |
|  | Gly_radical (Glycine radical) | SLBB (SLBB domain) |
|  | Glyco_hydro_1 (Glycoside hydrolase family 1) 4 | SNF (Sodium:neurotransmitter symporter) |
|  | Glyco_hydro_20 (Glycoside hydrolase family 20) | Sod_Fe_C (Superoxide dismutase) |
|  | Glyco_hydro_32N (Glycoside hydrolase family 32) | Sod_Fe_N (Superoxide dismutase) |
|  | Glyco_hydro_38 (Glycoside hydrolase family 38) | Sortase (Sortase) 3 |
|  | Glyco_hydro_88 (Glycoside hydrolase family 88) | SpoU_methylase (SpoU rRNA Methylase family) |
|  | Glyco_hydro_98C (Glycoside hydrolase family 98) | SSB (Single-strand binding protein family) |
|  | Glyco_hydro_98M (Glycoside hydrolase family 98) | Streptin-Immun (Lantibiotic streptin immunity protein) |
|  | Glyco_trans_4_2 (Glycosyl transferase 4-like) | Tautomerase (4-Oxalocrotonate tautomerase) |
|  | Glyco_transf_5 (Starch synthase catalytic domain) | TENA_THI-4 (TENA/THI-4/PQQC family) |
|  | Glyco_transf_8 (Glycosyl transferase family 8) 5 | TetR_N (Bacterial regulatory proteins, tetR family) 3 |
|  | Glycos_transf_1 (Glycosyl transferases group 1) 4 | Tex_N (Tex-like protein N-terminal domain) |
|  | Glycos_transf_2 (Glycosyl transferase family 2) 2 | ThiW (Thiamine-precursor transporter protein (ThiW)) |
|  | Glyoxalase (Glyoxalase/Bleomycin resistance protein/Dioxygenase superfamily) | Thr_dehydrat_C (C-terminal regulatory domain of Threonine dehydratase) |
|  | Glyoxalase_2 (Glyoxalase-like domain) | Thymidylate_kin (Thymidylate kinase) |
|  | GntR (GntR-like bacterial transcription factors) 2 | TipAS (TipAS antibiotic-recognition domain) |
|  | Gp49 (Phage derived protein Gp49-like (DUF891)) | TMP-TENI (Thiamine monophosphate synthase/TENI) 2 |
|  | Gram_pos_anchor (Gram positive anchor) | Transket_pyr (Transketolase, pyrimidine binding domain) |
|  | GSHPx (Glutathione peroxidase) | Transketolase_C (Transketolase, C-terminal domain) |
|  | GTP_cyclohydroI (GTP cyclohydrolase I) | Transketolase_N (Transketolase, thiamine diphosphate binding domain) |
|  | GTPase_Cys_C (Catalytic cysteine-containing C-terminus of GTPase, MnmE) | Trep_Strep (Hypothetical bacterial integral membrane protein (Trep_Strep)) |
|  | GtrA (GtrA-like protein) | TrkH (Cation transport protein) |
|  | Helicase_C (Helicase conserved C-terminal domain) 2 | TrmE_N (GTP-binding protein TrmE N-terminus) |
|  | Hepar_II_III (Heparinase II/III-like protein) | tRNA-synt_2d (tRNA synthetases class II core domain (F)) |
|  | HHH_3 (Helix-hairpin-helix motif) 2 | Trp_syntA (Tryptophan synthase alpha chain) |
|  | HK (Hydroxyethylthiazole kinase) | UPF0051 (Uncharacterized protein family (UPF0051)) |
|  | HsdM_N (DNA methyltransferase) | Usp (Universal stress protein family) |
|  | HTH_1 (Helix-turn-helix) | UTRA (UTRA domain) 2 |
|  | HTH_11 (Helix-turn-helix) 3 | V_ATPase_I (V-type ATPase 116kDa subunit family) 2 |
|  | HTH_19 (Helix-turn-helix) 5 | vATP-synt_AC39 (ATP synthase (C/AC39) subunit) |
|  | HTH_26 (Helix-turn-helix) | VWA (Von Willebrand factor type A domain) |
|  | HTH_3 (Helix-turn-helix) 4 | Wzz (Chain length determinant protein) |
|  | HTH_6 (Helix-turn-helix) | Y1_Tnp (Transposase IS200 like) |
|  | HTH_DeoR (Helix-turn-helix) | YajC (Preprotein translocase subunit) |
|  | HTH_Mga (Helix-turn-helix) 2 | YfhO (Bacterial membrane protein YfhO) |
|  | HTH_Tnp_ISL3 (Helix-turn-helix domain of transposase family ISL3) | YSIRK_signal (YSIRK type signal peptide) 3 |
|  | SSF (Sodium-solute symporter) | zf-CHY (CHY zinc finger) |
