# Supplementary material for: A functional genomics catalogue of activated transcription factors during pathogenesis of pneumococcal disease
Source: BMC Genomics. 2014 Sep 8;15(1):769. doi: 10.1186/1471-2164-15-769 (PMC4171566; doi:10.1186/1471-2164-15-769)
Supplement: Supplementary file 13 — Additional file 13: Table S12: Primers for construction of mutants, cloning, sequencing and real-time RT-PCR analysis. (DOCX 85 KB) [file 12864_2014_6462_MOESM13_ESM.docx]

**Table S12.** Primers for construction of mutants, cloning, sequencing and real-time RT-PCR analysis.

**Primers for construction and validation of mutants.**

| **Primer Name** | **Sequence 5’ to 3’** |
| --- | --- |
| CodY Flank F | GCTCTTAAATCTGGTGCCCATATTGT |
| CodY Flank R | CGATGGATATGTTTAGTCCTTACT |
| CodY Ery X | TTGTTCATGTAATCACTCCTTCTTAATCGCTATCATTATAACATAAAAAC |
| CodY Ery Y | CGGGAGGAAATAATTCTATGAGCTTTAATTTCGATTGATTATACAGAAG |
| CodY Spec X | TATGTATTCATATATATCCTCCTCTTTAATCGCTATCATTATAACATAAA |
| CodY Spec Y | AAATAACAGATTGAAGAAGGTATAATGACAAAGGCTTTAATTTCGATTGA |
| CodY UpSeq | GTGTTATACTAGATAGGTTGCAAAG |
| AmiC F | CTCTCCATGAGGAGAAGGTTCT |
| AmiC R | CAAAATCGTCACGTTTTACAAACTG |
| FatC F | CTAGGAATCATCGGTAGTCTCG |
| FatC R | GCAAAATATCCTGTTTTTGAATGGA |

**Primers for cloning, sequencing and real-time RT-PCR analysis**.

| **Primer Name** | **Sequence 5’ to 3’** |
| --- | --- |
| CodY Clone F | TGATAGCGATTGAATTCGAGGTGAA |
| CodY Clone R | ATCAATCGAATTCAAAGCCTTTGTCATTAG |
| CodY Seq F | AGGCAACTTGTCAATAGAAAAGGAAC |
| CodY Seq R | GTAAAGAAGATATAATCTCCTCGTTC |
| CodY F | TTCGTCGTCGTACTGCTGTC |
| CodY R | CTTTCAATAATCCCCGCAGA |
